# Association of the TyG index with prognosis in surgical intensive care patients: data from the MIMIC-IV

**DOI:** 10.1186/s12933-024-02293-0

**Published:** 2024-06-06

**Authors:** Donghao Liu, Bingkui Ren, Yuqing Tian, Zhigang Chang, Tong Zou

**Affiliations:** 1 Department of Cardiology, Beijing Hospital, National Center of Gerontology, Institute of Geriatric Medicine, Chinese Academy of Medical Sciences, Beijing, 100730 People’s Republic of China; 2 Beijing Hospital, National Center of Gerontology, Institute of Geriatric Medicine, Chinese Academy of Medical Sciences & Peking Union Medical College, Beijing, 100730 People’s Republic of China; 3 Beijing Hospital, Institute of Geriatric Medicine, Peking University Fifth School of Clinical Medicine, Beijing, People’s Republic of China; 4 Department of Critical Care Medicine, Beijing Hospital, National Center of Gerontology, Institute of Geriatric Medicine, Chinese Academy of Medical Sciences, Beijing, 100730 People’s Republic of China

**Keywords:** MIMIC-IV database, Insulin resistance, Surgery, Trauma, Triglyceride–glucose index, Prognosis

## Abstract

**Background:**

The triglyceride–glucose (TyG) index, a tool for assessing insulin resistance, is increasingly recognized for its ability to predict cardiovascular and metabolic risks. However, its relationship with trauma and surgical patient prognosis is understudied. This study investigated the correlation between the TyG index and mortality risk in surgical/trauma ICU patients to identify high-risk individuals and improve prognostic strategies.

**Methods:**

This study identified patients requiring trauma/surgical ICU admission from the Medical Information Mart for Intensive Care (MIMIC-IV) database, and divided them into tertiles based on the TyG index. The outcomes included 28-day mortality and 180-day mortality for short-term and long-term prognosis. The associations between the TyG index and clinical outcomes in patients were elucidated using Cox proportional hazards regression analysis and RCS models.

**Results:**

A total of 2103 patients were enrolled. The 28-day mortality and 180-day mortality rates reached 18% and 24%, respectively. Multivariate Cox proportional hazards analysis revealed that an elevated TyG index was significantly related to 28-day and 180-day mortality after covariates adjusting. An elevated TyG index was significantly associated with 28-day mortality (adjusted hazard ratio, 1.19; 95% confidence interval 1.04–1.37) and 180-day mortality (adjusted hazard ratio, 1.24; 95% confidence interval 1.11–1.39). RCS models revealed that a progressively increasing risk of mortality was related to an elevated TyG index. According to our subgroup analysis, an elevated TyG index is associated with increased risk of 28-day and 180-day mortality in critically ill patients younger than 60 years old, as well as those with concomitant stroke or cardiovascular diseases. Additionally, in nondiabetic patients, an elevated TyG index is associated with 180-day mortality.

**Conclusion:**

An increasing risk of mortality was related to an elevated TyG index. In critically ill patients younger than 60 years old, as well as those with concomitant stroke or cardiovascular diseases, an elevated TyG index is associated with adverse short-term and long-term outcomes. Furthermore, in non-diabetic patients, an elevated TyG index is associated with adverse long-term prognosis.

**Supplementary Information:**

The online version contains supplementary material available at 10.1186/s12933-024-02293-0.

## Introduction

The triglyceride–glucose index (TyG) is a tool for indirectly assessing insulin resistance (IR) by using a mathematical model based on fasting plasma triglyceride and blood glucose levels [1]. In recent years, this index has garnered increased attention due to its simplicity, cost-effectiveness [2], and its ability to predict for various cardiovascular and metabolic risks and conditions [3]. Many studies have found that an elevated TyG index is associated with cardiovascular events in specific populations [4, 5], and this conclusion has been confirmed in numerous large databases [6–9]. Insulin resistance is one of the most basic responses to injury and stress [10], and in major surgeries such as large bowel surgeries, up to 90% of insulin sensitivity may be lost postoperatively [11]. However, the specific relationship between the TyG index, as an indirect indicator of insulin resistance, and the prognosis of trauma and surgical patients has been mentioned in relatively few literature. This study aims to investigate the correlation between the TyG index and the short-term and long-term mortality risks of patients admitted to surgical ICU/trauma ICU, which may help us identify high-risk patients and potentially contribute to the development of new strategies to improve patient prognosis.

## Methods

### Data source

Data was acquired from the Multiparameter Intelligent Monitoring in Intensive Care Database IV (MIMIC-IV) version 2.2 which enrolled more than 70,000 ICU stays between 2008 and 2019 [12]. The database was operated by the Beth Israel Deaconess Medical Center. We accomplish the course, Protecting Human Research Participants (certification number: 46538344), which is a National Institutes of Health web-based course. Our permission was approved by the Institutional Review Boards of the Massachusetts Institute of Technology (Cambridge, MA, USA) and the Beth Israel Deaconess Medical Center.

### Population selection

**Fig. 1:**
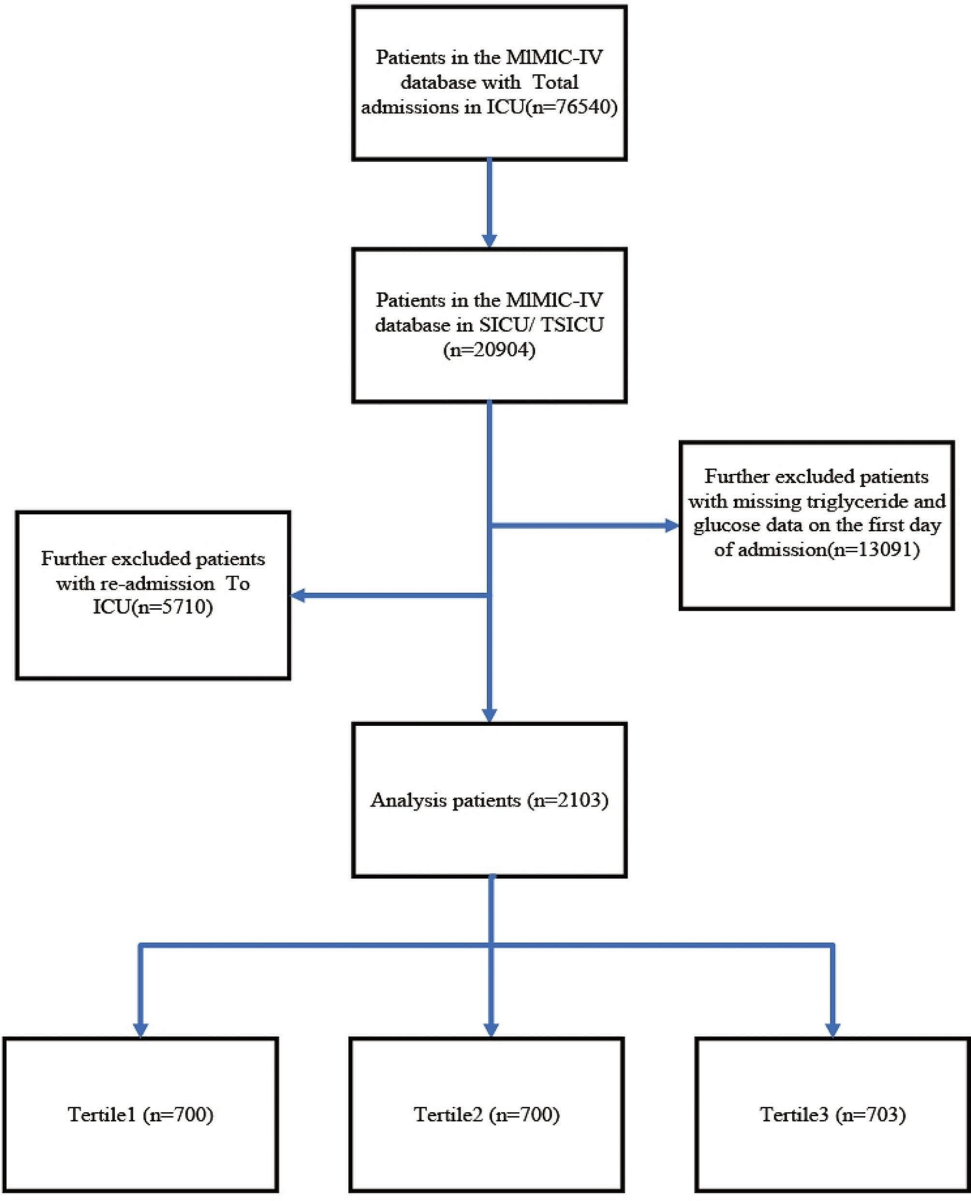
Flow chart of study participants

Patients were enrolled if they met the following criteria: (1) admitted to the surgical intensive care unit (ICU) or trauma surgical ICU(TSICU). (2) Patients for whom the TyG index could be calculated. (3) aged ≥ 18 years; (4) stayed in the ICU for more than 24 h; Finally, a total of 2103 patients were enrolled in this study and grouped into three groups based on the tertiles of the TyG index, please refer to Figure 1 for the specific research process.

### Data extraction

We utilized structured query language (SQL) to acquire information recorded on the first day after ICU admission. The sex, race and age of the patients; the Simplified Acute Physiology Score (SAPS) II and the sequential organ failure assessment (SOFA) score, laboratory tests including white blood cells (WBC), serum creatine (Scr), hemoglobin, platelets, comorbidities including heart failure (HF), hypertension, chronic obstructive pulmonary disease (COPD), chronic kidney disease (CKD), atrial fibrillation (AF), stroke, diabetes, cardiovascular disease. All these data stated above were extracted and can be seen in Table 1. We considered 28-day mortality to be the indicator of short-term prognosis, and 180-day mortality to be the indicator of long-term prognosis. All laboratory variables were obtained only within the first 24 h after patient admission, excluding variables with missing values ​​exceeding 20% to mitigate potential biases. For variables with less than 20% missing values, multiple imputation was performed using the random forest imputation method implemented in the mice package of R software [13].

**Table 1:** Characteristics and outcomes of participants categorized by TyG index

Characteristic		TyG index											
		Overall, *N* = 2103^a^			T1 *N* = 700^a^		T2 *N* = 700^a^		T3 *N* = 703^a^		*p*-value^b^		q-value^c^
TyG index		9.20 (0.84)			8.38 (0.32)		9.07 (0.19)		10.13 (0.66)		**< 0.001**		< 0.001
Fasting blood glucose (mg/dl)		166 (89)			125 (36)		157 (55)		217 (123)		<**0.001**		< 0.001
Triglyceride (mg/dl)		180 (233)			76 (23)		123 (40)		340 (348)		<**0.001**		< 0.001
First careunit											**< 0.001**		< 0.001
Surgical intensive care unit (SICU)		1406 (67%)			504 (72%)		480 (69%)		422 (60%)				
Trauma SICU (TSICU)		697 (33%)			196 (28%)		220 (31%)		281 (40%)				
Age (years)		64 (17)			69 (18)		66 (17)		58 (16)		**< 0.001**		< 0.001
Race											**< 0.001**		0.031
Asian		60 (2.9%)			20 (2.9%)		15 (2.1%)		25 (3.6%)				
Black		176 (8.4%)			66 (9.4%)		53 (7.6%)		57 (8.1%)				
Other		535 (25%)			148 (21%)		171 (24%)		216 (31%)				
White		1332 (63%)			466 (67%)		461 (66%)		405 (58%)				
Weight (kg)		83 (24)			76 (21)		82 (23)		90 (25)		**< 0.001**		< 0.001
Unknown		14			5		2		7				
Sex											**< 0.001**		0.008
Female		924 (44%)			330 (47%)		329 (47%)		265 (38%)				
Male		1179 (56%)			370 (53%)		371 (53%)		438 (62%)				
APSIII (median [IQR])		39 [29, 54]			34 [26, 46]		38 [28, 54]		45 [32, 64]		**< 0.001**		< 0.001
SAPSII (median [IQR])		33 [25, 43]			31 [24, 38]		33 [25, 42]		36 [27, 47]		**< 0.001**		< 0.001
OASIS (median [IQR])		32 [26, 38]			30 [25, 36]		32 [27, 37]		34 [28, 40]		**< 0.001**		< 0.001
SOFA (median [IQR])		4 [2, 6]			3 [1, 4]		3 [2, 6]		5 [3, 9]		**< 0.001**		< 0.001
Commorbidities													
Unknown		1			0		0		1				
Heart failure		301 (14%)			108 (15%)		102 (15%)		91 (13%)		0.4		> 0.9
Hypertension		1392 (66%)			475 (68%)		489 (70%)		428 (61%)		**0.001**		0.035
Arterial fibrillation		429 (20%)			169 (24%)		165 (24%)		95 (14%)		**< 0.001**		< 0.001
Diabetes		495 (24%)			87 (12%)		175 (25%)		233 (33%)		**< 0.001**		< 0.001
Renal disease		263 (13%)			75 (11%)		86 (12%)		102 (15%)		0.095		> 0.9
Liver disease		150 (7.1%)			32 (4.6%)		51 (7.3%)		67 (9.5%)		**0.001**		0.045
COPD		221 (11%)			79 (11%)		63 (9.0%)		79 (11%)		0.3		> 0.9
Cardiovascular disease		341 (16%)			114 (16%)		128 (18%)		99 (14%)		0.10		> 0.9
Stroke		1124 (53%)			451 (64%)		422 (60%)		251 (36%)		**< 0.001**		< 0.001
Maligancy tumour		296 (14%)			84 (12%)		88 (13%)		124 (18%)		**0.004**		0.11
SBP (/mmHg)											**< 0.001**		< 0.001
< 90		65 (3.1%)			17 (2.4%)		19 (2.7%)		29 (4.2%)				
90–140		1189 (57%)			379 (54%)		366 (52%)		444 (64%)				
> 140		839 (40%)			301 (43%)		315 (45%)		223 (32%)				
Unknown		10			3		0		7				
DBP (/mmHg)											0.11		> 0.9
≤ 60		513 (25%)			161 (23%)		162 (23%)		190 (27%)				
> 60		1580 (75%)			536 (77%)		538 (77%)		506 (73%)				
Unknown		10			3		0		7				
Laboratory tests													
eGFR (ml/min)		78 (31)			81 (28)		77 (30)		74 (34)		**0.008**		0.3
Unknown		22			6		10		6				
WBC, (K/µL)		11.6 (6.1)			10.3 (4.9)		11.6 (6.0)		12.8 (7.0)		**< 0.001**		< 0.001
Unknown		28			10		11		7		28		
Hemoglobin (g/dL)		11.80 (2.25)			11.97 (2.04)		11.80 (2.33)		11.61 (2.36)		**0.007**		0.2
Unknown		29			11		10		8		29		
Platelet (K/µL)		215 (100)			217 (97)		221 (102)		207 (101)		**0.016**		0.5
Unknown		27			10		11		6				
INR		1.41 (0.87)			1.40 (0.87)		1.37 (0.61)		1.45 (1.06)		0.061		> 0.9
Unknown		346			135		116		95				
28-Day death		375 (18%)			120 (17%)		134 (19%)		121 (17%)		0.5		> 0.9
180-Day death		559 (27%)			170 (24%)		204 (29%)		185 (26%)		0.12		> 0.9
ICU-LOS (days)		8 (10)			4 (7)		7 (10)		11 (12)		**< 0.001**		< 0.001
LOS (days)		14 (15)			10 (9)		13 (15)		19 (19)		**< 0.001**		< 0.001

The bold part indicates that P < 0.05, which is statistically significant

^a^Mean (SD); n (%)

^b^Kruska–Wallis rank sum test; Pearson’s Chi-squared test

^c^Bonferroni correction for multiple testing

### Statistical analysis

Continuous variables, are presented as the mean and standard deviation (SD), while categorical variables are presented as counts and percentages. The Kruskal-Wallis rank sum test is used for continuous variables, and chi-squared test is used for categorical variables. To explore the relationships between variables and all-cause mortality, we conducted event-time analysis. We validated the proportional hazards assumption of variables using Schoenfeld residuals. Single-factor and multifactor analyses were performed using Cox proportional hazards regression models. The Tyg index was calculated based on triglyceride (TG) and fasting blood glucose (FBG) levels with the following formula:

$$TyG\, Index=\text{ln}\left[TG\left({\text{mg}}/{\text{dl}}\right)\right.\times \left[FBG\left({\text{mg}}/{\text{dl}}\right)/2\right]$$

The TyG index was divided into three groups according to the tertiles of the target patient population: T1 (< 8.76), T2 (8.76–9.43), and T3 (> 9.43). Adjustments were made in the model for demographic characteristics and comorbidity-related variables, including age (as a continuous variable), race and ethnicity (White, Black, Asian, Other), sex, weight (as a continuous variable), eGFR (CKD-EPI [14]), heart failure, hypertension, diabetes, acute kidney injury, acute liver injury, COPD, coronary artery disease, and stroke. To address the issue of multiple testing, the Benjamini–Hochberg procedure was applied to control the false discovery rate (FDR). The median of each group was used as the continuous variable in the model for trend testing. To explain multicollinearity, variance inflation factors (VIFs) were calculated to assess the multicollinearity of variables in the multivariable Cox model. A VIF < 10 indicates that multicollinearity may not affect the estimation [15]. Additionally, stratified analyses were conducted based on different demographic characteristics and comorbidities, with subgroup and TyG index (T1, T2, T3) multiplicative interaction terms fitted into the model to evaluate potential interaction effects.

In the model, the TyG index was included as a continuous variable, and the restricted cubic spline (RCS) curve was analyzed as a continuous variable to elucidate the relationship between dose effects and the risks of both short-term and long-term outcome. At the same time, subgroups where interactions were found in stratified analysis are further studied by incorporating them into the generalized additive models. All analyses were conducted using R software (version 4.3.0). A *p* value less than 0.05 was considered statistically significant.

## Result

### Baseline

A total of 2103 patients admitted to the Surgical Intensive Care Unit (SICU) were included in our study. The 28-day in-hospital mortality for the included patients was 18%, and the 180-day mortality was 27%. According to Table 1, with the T1 group as the reference, as the TyG index increases, the likelihood of admission to Trauma SICU increases, age tends to be younger, the level of fasting blood glucose and triglyceride tends to be higher, weight tends to be higher, and the likelihood of being male increases. The severity of illness score upon admission is higher, and the prevalence of atrial fibrillation and stroke is lower. The proportion of patients with shock (SBP < 90) and diabetes is higher. The ICU length of stay and hospitalization days are longer.

### Associations between the TyG index and all-cause mortality

As shown in Table 2, when TyG is treated as a categorical variable, after adjustment for age, sex, race/ethnicity, comorbidities, and eGFR (Model 3), compared to T1, the 28-day mortality HR (95% CI) for T2 is 1.22 (0.95, 1.57), and the 180-day mortality HR (95% CI) is 1.36 (1.11, 1.68). The 28-day mortality HR (95% CI) for T3 is 1.32 (0.99, 1.76), and the 180-day mortality HR (95% CI) is 1.41 (1.12, 1.79). For each 1-unit increase in TyG, the change in 28-day HR (95% CI) is 1.19 (1.04, 1.37), and the change in 180-day HR (95% CI) is 1.24 (1.11, 1.39). The trend test for 180-day mortality is statistically significant.

**Table 2:** Cox proportional hazard ratios (HR) for all-cause mortality

Models	TyG index				TyG index increase 1 unit	*P* for trend
	T1 (< 8.76)		T2 (8.76–9.43)	T3 (> 9.43)		
28-Day mortality						
Model 1 HR (95% CI)	Ref		1.13 (0.89, 1.45)	1.01 (0.78, 1.30)	**1.03 (0.92, 1.16)**	0.96
Model 2 HR (95% CI)	Ref		1.27 (0.99, 1.63)	**1.41 (1.08, 1.83)**	**1.23 (1.09, 1.39)**	**0.009**
Model 3 HR (95% CI)	Ref		1.22 (0.95, 1.57)	1.32 (0.99, 1.76)	**1.19 (1.04, 1.37)**	0.051
180-Day mortality						
Model 1 HR (95% CI)	Ref		1.23 (1.00, 1.51)	1.09 (0.89, 1.34)	**1.08 (0.99, 1.19)**	0.43
Model 2 HR (95% CI)	Ref		**1.39 (1.13, 1.70)**	**1.53 (1.23, 1.90)**	**1.29 (1.16, 1.42)**	**< 0.001**
Model 3 HR (95% CI)	Ref		**1.36 (1.11, 1.68)**	**1.41 (1.12,1.79)**	**1.24 (1.11, 1.39)**	**0.003**

The bold part indicates that P < 0.05, which is statistically significant

Model 1: Non-adjusted

Model 2: Adjusted for age, sex, race/ethnicity

Model 3: Adjusted for age, sex, race/ethnicity, heart failure, hypertension, arterial fibrillation, diabetes, renal disease, liver disease, COPD, cardiovascular disease, stroke, eGFR

#### Dose–response analysis of the TyG index and all-cause mortality

**Fig. 2:**
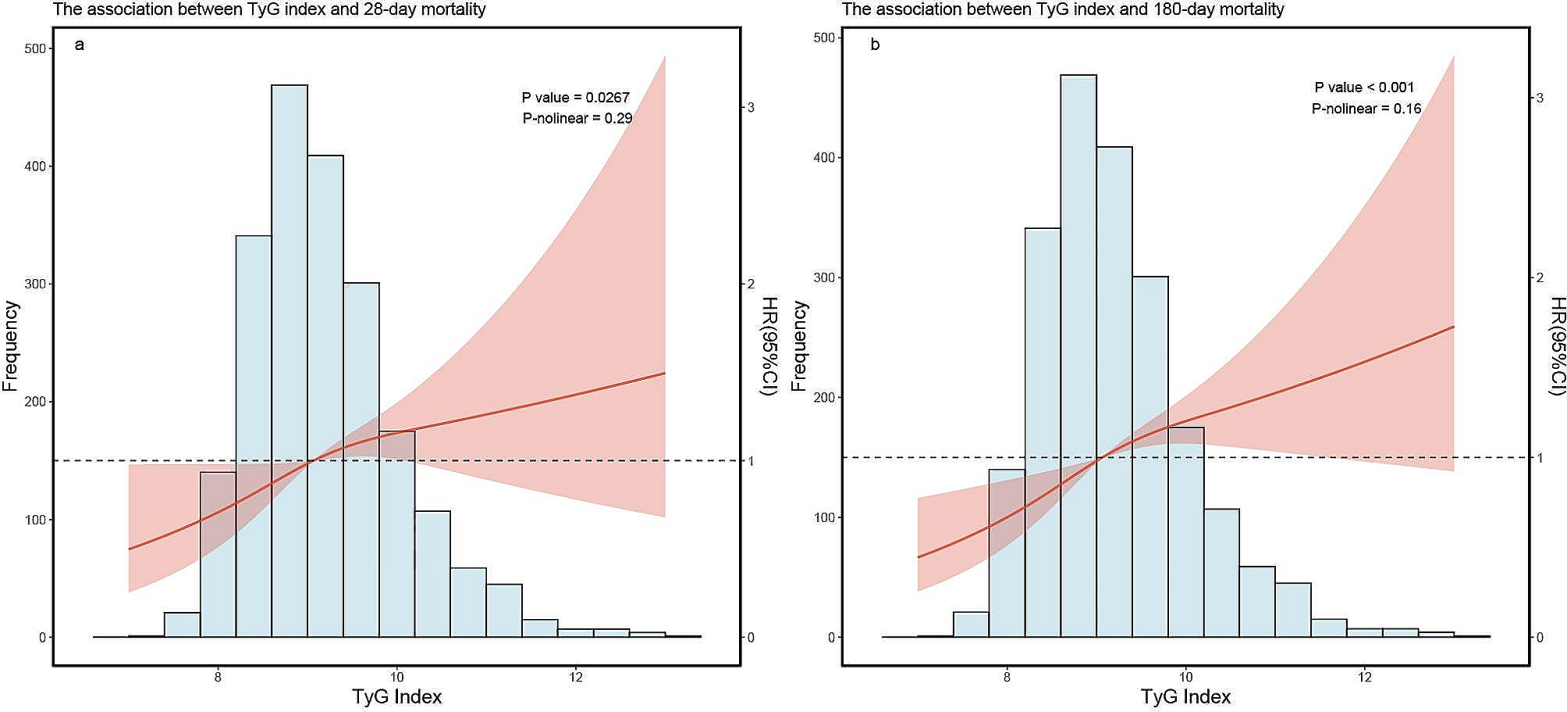
RCS curve for the TyG index hazard ratio. The solid line and the ribbon represent the estimated HRs and its 95% CI with median serving as the reference point. **a** Represent the curve in 28-day mortality, **b** represent the curve in 180-day mortality. All model adjusted for age, sex, race/ethnicity, comorbidities. RCS, restricted cubic spline; HR, hazard ratio; CI, confidence interval; TyG, triglyceride–glucose

After adjusting for age, sex, race/ethnicity, comorbidities, and eGFR, RCS curve analysis reveals a linear correlation between the TyG index and 28-day mortality as well as 180-day mortality (*p* value < 0.05, p for nonlinear > 0.05) (See Fig. 2).

### Subgroup analysis

After adjusting for all covariates, the subgroup analysis results are as follows (Supplemental Figs. 1, 2, 3, 4). Regarding 28-day mortality, compared to T1 as reference, there are differences in the impact of T2 on 28-day mortality in the subgroups of stroke and heart failure; the impact of T3 on 28-day mortality differs in subgroups of age, race, stroke, atrial fibrillation, and types of ICU, with an interaction between ICU type and this variable (*P* < 0.05). Regarding 180-day mortality, compared to T1 as reference, the impact of T2 on 180-day mortality differs in subgroups of gender, age, race, hypertension, stroke, atrial fibrillation, heart failure, and ICU; the impact of T3 on 180-day mortality differs in subgroups of gender, age, race, coronary heart disease, stroke, atrial fibrillation, heart failure, and ICU type, with interactions between age, stroke, and this variable (*P* < 0.05).

### Dose–response curve in subgroup

#### Age

**Fig. 3:**
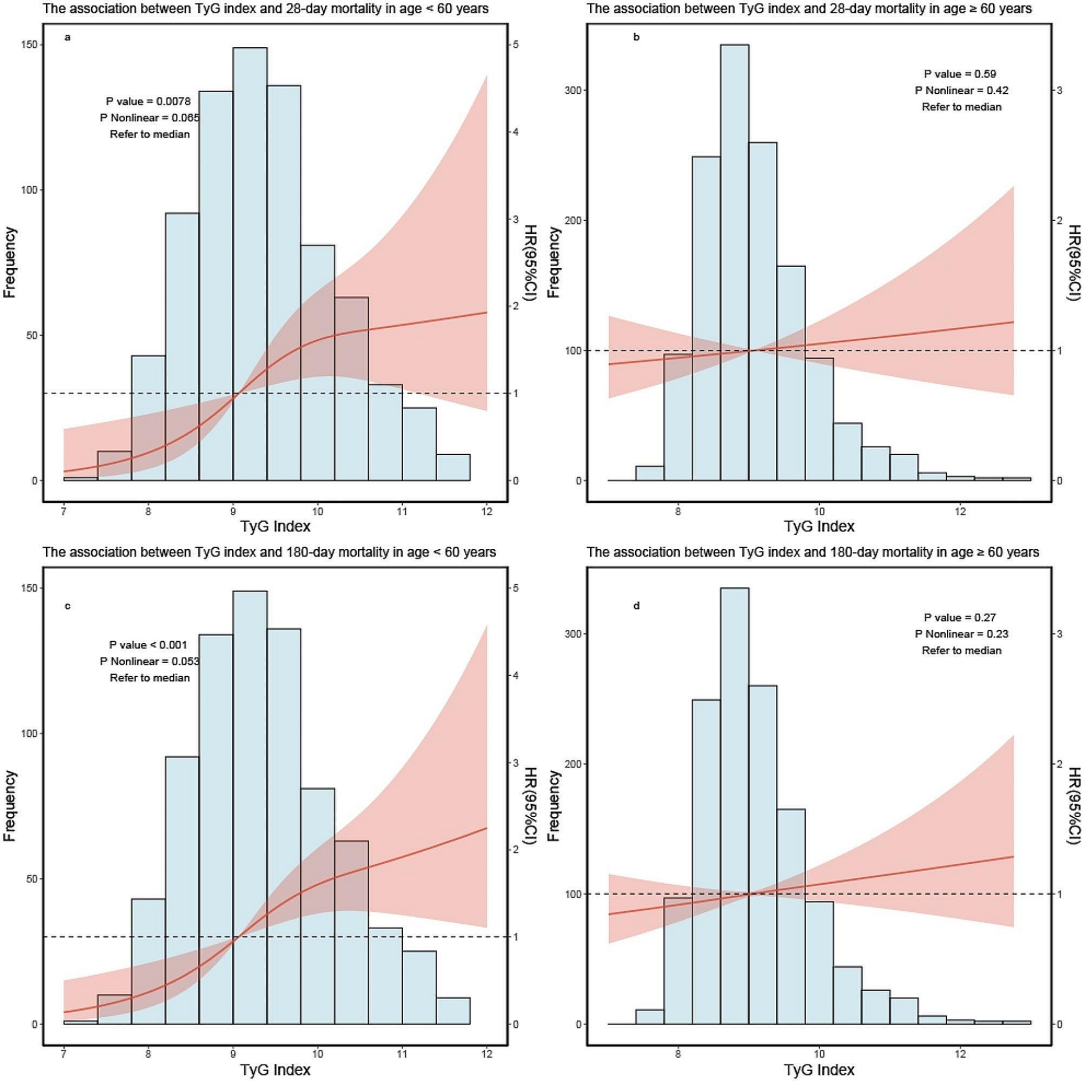
RCS curve for the TyG index hazard ratio in subgroup analysis. The solid line and the ribbon represent the estimated HRs and its 95% CI with median serving as the reference point. **a** Represent the curve in age < 60 years in 28-day mortality, **b** represent the curve in age ≥ 60 years in 28-day mortality. **c** Represent the curve in age < 60 years in 180-day mortality, **d** represent the curve in age ≥ 60 years in 180-day mortality. RCS, restricted cubic spline; HR, hazard ratio; CI, confidence interval; TyG, triglyceride–glucose

In the subgroup dose–response curve, after adjusted for all covariates (similar to covariates in the dose–response) except for the subgroups, we found that when 28 days was used as the endpoint outcome time, TyG index was correlated with 28-day mortality in patients under 60 years old, while it was not correlated in patients aged 60 or elder (Fig. 3a, b). When 180 days was used as the endpoint outcome time, TyG index was positively correlated with 180-day mortality in patients under 60 years old, while it was not correlated in patients aged 60 or elder (Fig. 3c, d).

#### Diabetes

**Fig. 4:**
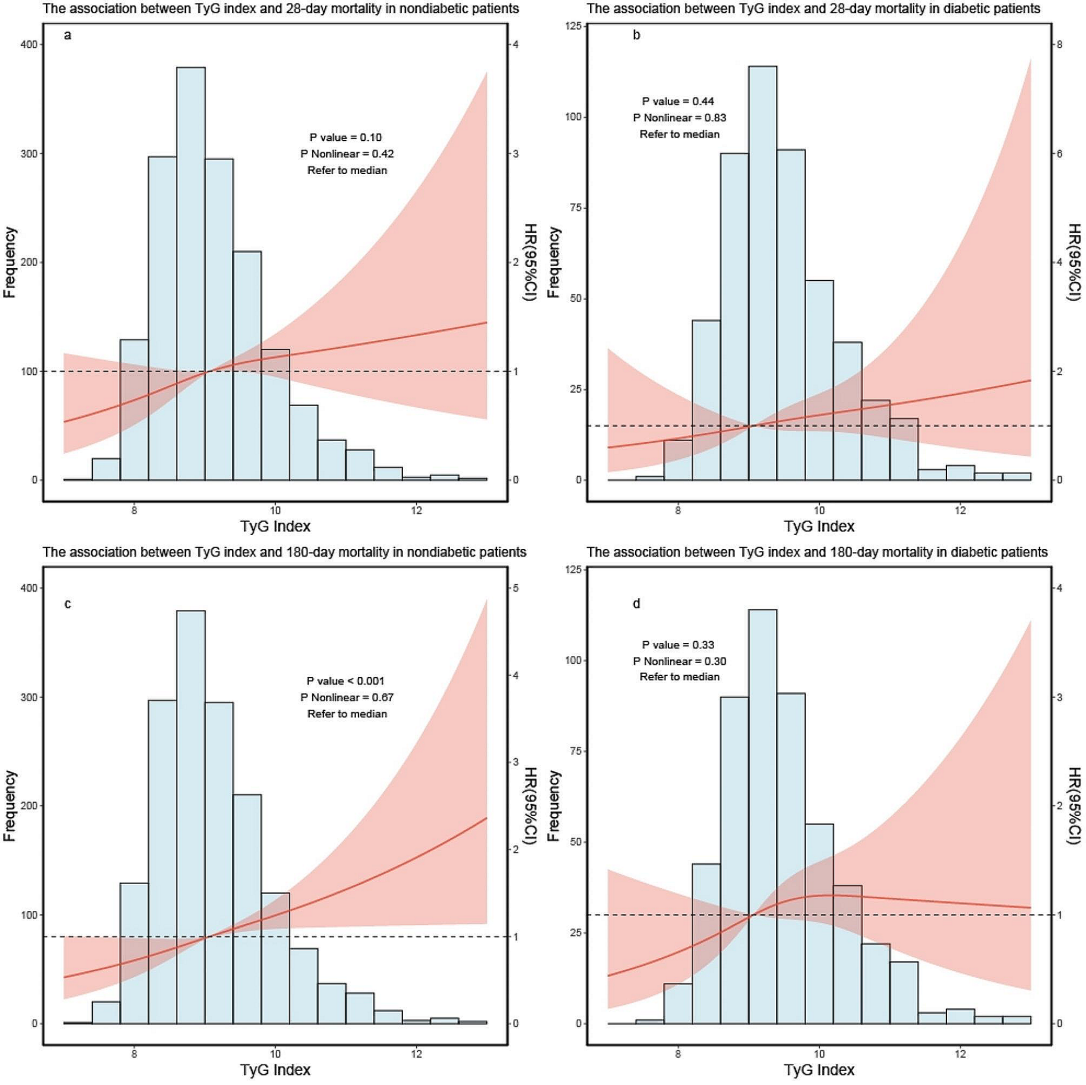
RCS curve for the TyG index hazard ratio in subgroup analysis. The solid line and the ribbon represent the estimated HRs and its 95% CI with median serving as the reference point. **a** Represent the curve in nondiabetic patients in 28-day mortality, **b** represent the curve in diabetic patients in 28-day mortality. **c** Represent the curve in nondiabetic patients in 180-day mortality, **d** represent the curve in diabetic patients in 180-day mortality. RCS, restricted cubic spline; HR, hazard ratio; CI, confidence interval; TyG, triglyceride–glucose

After adjusted for all covariates (similar to covariates in the dose–response) except for the subgroups, when 28 days was used as the endpoint outcome time, TyG and mortality were not correlated in both diabetic and nondiabetic patients (Fig. 4a, b), when 180 days was used as the endpoint outcome time, TyG index was positively correlated with 180-day mortality rate in nondiabetic patients, while it was not correlated in diabetic patients (Fig. 4c, d).

#### Stroke

**Fig. 5:**
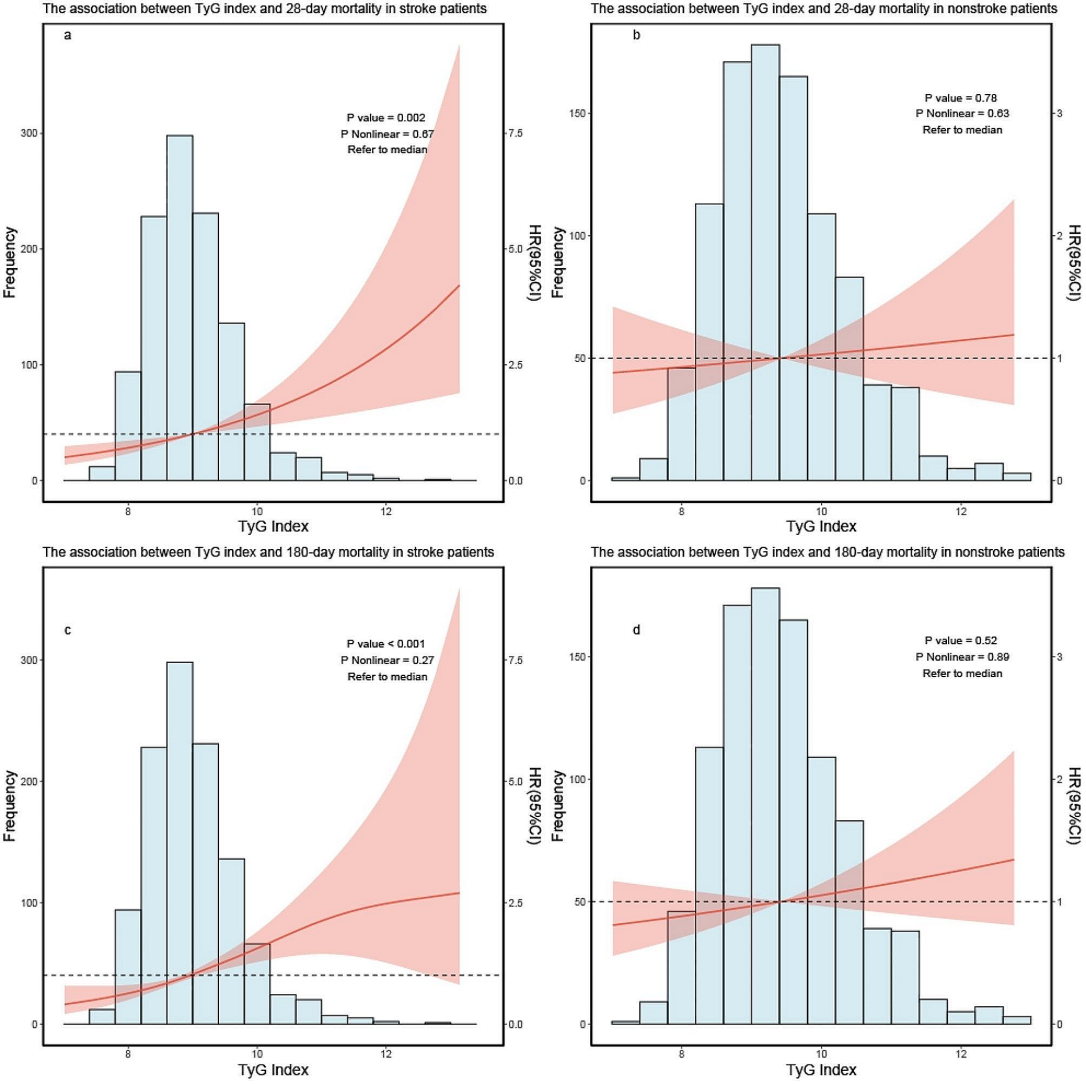
RCS curve for the TyG index hazard ratio in subgroup analysis. The solid line and the ribbon represent the estimated HRs and its 95% CI with median serving as the reference point. **a** Represent the curve in stroke patients in 28-day mortality, b represent the curve in nonstroke patients in 28-day mortality. **c** Represent the curve in stroke patients in 180-day mortality, **d** represent the curve in nonstroke patients in 180-day mortality. RCS, restricted cubic spline; HR, hazard ratio; CI, confidence interval; TyG, triglyceride–glucose

After adjusted for all covariates (similar to covariates in the dose–response) except for the subgroups, when 28 days was used as the endpoint outcome time, TyG index was positively correlated with 28-day mortality in stroke patients, while it was not correlated in nonstroke patients (Fig. 5a, b), when 180 days was used as the endpoint outcome time, TyG index was positively correlated with 180-day mortality in stroke patients, while it was not correlated in nonstroke patients (Fig. 5c, d).

#### Cardiovascular disease (Cad)

**Fig. 6:**
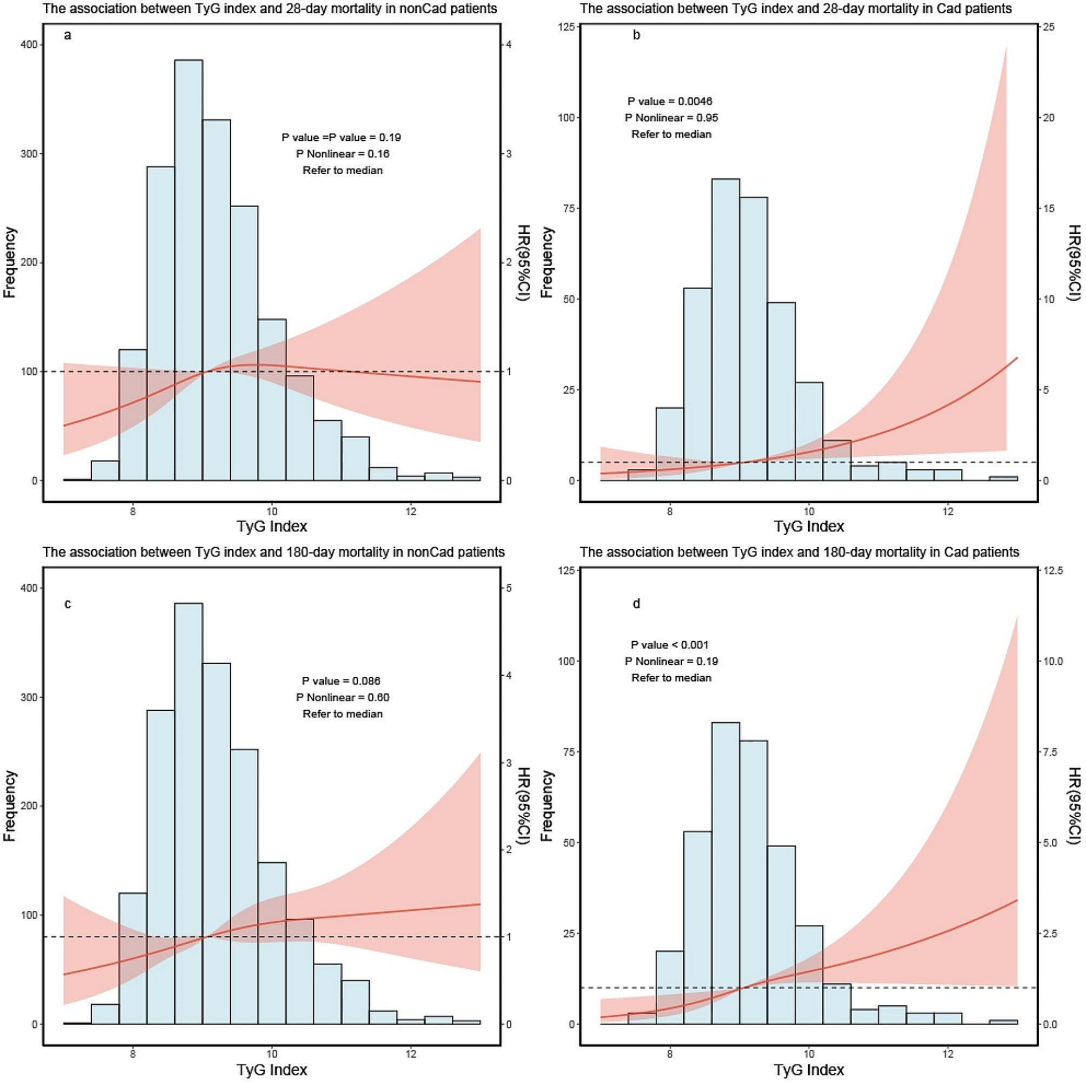
RCS curve for the TyG index hazard ratio in subgroup analysis. The solid line and the ribbon represent the estimated HRs and its 95% CI with median serving as the reference point. **a** Represent the curve in nonCad patients in 28-day mortality, **b** represent the curve in Cad patients in 28-day mortality. **c** Represent the curve in nonCad patients in 180-day mortality, **d** represent the curve in Cad patients in 180-day mortality. RCS, restricted cubic spline; HR, hazard ratio; CI, confidence interval; TyG, triglyceride–glucose; Cad, Cardiovascular disease

After adjusted for all covariates (similar to covariates in the dose–response) except for the subgroups, when 28 days was used as the endpoint outcome time, TyG index was positively correlated with 28-day mortality in patients with Cad, while it was not correlated in patients without Cad (Fig. 6a, b), when 180 days was used as the endpoint outcome time, TyG index was positively correlated with 180-day mortality in patients with Cad, while it was not correlated in patients without Cad (Fig. 6c, d).

## Discussion

To investigate whether insulin resistance induced by severe trauma and resulting physiological stress affects patient mortality, we selected all patients in the surgical ICU (including trauma ICU) in MIMIC-IV to study the relationship between the Tyg index and prognosis, to indirectly reflect the degree of insulin resistance through the TyG index and established its relationship with prognosis. This study demonstrates for the first time the correlation between the TyG index and short-term and long-term prognosis in the surgical ICU. Our research indicates that, in general, the TyG index is positively correlated with the short-term and long-term mortality rates of surgical critically ill patients. Subgroup analysis shows that in patients under 60 years old, patients with Cad, and patients with stroke, an increase in the TyG index is associated with adverse short-term and long-term outcomes. In addition, in non-diabetic patients, an increase in the TyG index is associated with adverse long-term outcomes, but not with short-term outcomes. Among these, the results we found in the stroke and coronary heart disease populations are similar to previous research findings [16–19]. 

Existing research indicates that insulin resistance can lead to the occurrence of dyslipidemia triad (decreased high-density lipoprotein, increased low-density lipoprotein, increased triglycerides), thereby promoting endothelial dysfunction, local release of chronic inflammatory factors, endothelial damage, and the occurrence of atherosclerosis [20]. Firstly, when blood glucose levels rise, the transcription of angiotensinogen, angiotensin-converting enzyme (ACE), and angiotensin II in blood vessels will all increase significantly [21]. This will cause abnormal activation of the renin-angiotensin-aldosterone system (RAAS), and in conjunction with hyperinsulinemia, stimulate the activation of the MAPK pathway [22]. Simultaneously, when the body is in a state of insulin resistance, insulin-stimulated nitric oxide (NO) synthesis is inhibited. This prevents effective suppression of the MAPK pathway activation caused by hyperinsulinemia, thereby exacerbating its downstream effects (enhanced vasoconstriction, intensified local inflammatory response, etc.). This suggests that insulin resistance will disrupt the balance between endothelial vasoconstriction and dilation, thereby increasing cardiovascular risk [23]. A study from Korea suggests that the TyG index, which serves as an indicator of the degree of insulin resistance in the body, is associated with subclinical atherosclerosis. This association may be precisely due to the effects of the mechanisms mentioned above [24]. Furthermore, chronic caloric surplus associated with insulin resistance leads to an increase in visceral fat accumulation within the body [25]. As the severity of obesity increases, adipocytes release large amounts of chemokines, prompting monocytes to differentiate into macrophages and migrate to visceral adipose tissue (VAT). Subsequently, macrophages secrete significant amounts of TNFα, reducing the storage of insulin-related substances such as glucose transporter 4 and triglycerides in VAT. This leads to elevated circulating triglyceride levels, promoting ectopic deposition of toxic fatty acids in extraneous fat tissues (pancreas, kidneys, liver, skeletal muscle, heart, etc.), which is also a significant factor contributing to the increase in epicardial adipose tissue [26]. In a study involving patients with chronic coronary syndrome (CCS), it was found that patients with concomitant diabetes mellitus had significantly more volumes of epicardial adipose tissue compared to those without diabetes mellitus. Among CCS patients without concomitant diabetes mellitus, an increase in TyG index was associated with a significant increase in the risk of major adverse cardiovascular events (MACE) [27]. This study findings provide some insights: (1) Patients with CCS and concomitant diabetes mellitus may significantly improve ectopic lipid deposition through lifestyle and pharmacological interventions. However, these interventions may not completely eliminate the cardiovascular risk associated with irreversible insulin resistance caused by DM. (2) CCS patients without DM but with an elevated TyG index may require active intervention and dynamic monitoring of improvements in insulin resistance, which could lead to potential clinical benefits. (3) In addition to monitoring changes in the TyG index, timely assessment of epicardial adipose tissue volume is crucial for evaluating improvements in insulin resistance, as it is determined by the abnormal lipid deposition resulting from insulin resistance.

Insulin resistance, besides serving as the initiating factor for chronic metabolic-related diseases, can also be a product of stress. When the body is under stress (such as major illness or surgery), the nervous system activates the stress response by sending impulses to the hypothalamus. The hypothalamus either removes its inhibitory effect on the pituitary gland or releases hormones that stimulate pituitary hormone production and/or release. Pituitary hormones act on various target organs, promoting the release of effector hormones such as glucagon, catecholamines, and cortisol. These hormones facilitate glycogenolysis, gluconeogenesis, and lipolysis, leading to a significant increase in substances such as glucose and triglycerides in the peripheral blood [28]. Simultaneously, the activation of the body’s inflammatory response leads to the release of large amounts of immune cell cytokines (such as TNF-α, IL-1), thereby promoting the development of insulin resistance. This also suggests that the TyG index, besides serving as a marker of insulin resistance in the body, may also reflect the degree of immune inflammatory activation in the body [29]. A prospective study [30] suggests that combining optical coherence tomography (OCT) with the TyG index can effectively stratify the risk and predict the clinical prognosis of patients with ST-elevation myocardial infarction (STEMI). In this study, the TyG index showed a strong association with adverse clinical outcomes in patients with plaque rupture (PR), but no such association was found in patients with plaque erosion (PE). This indicates a significant correlation between the TyG index and plaque characteristics. It is well-known that compared to PE, PR is more unstable. This instability is associated with the thickness of the fibrous cap [31], the size of the lipid core [32], and the extent of local inflammatory infiltration [19]. As insulin resistance increases, ectopic lipid deposition becomes more pronounced, potentially leading to larger lipid cores in plaques and exacerbating local inflammatory responses [19]. In response, inflammatory cells secrete various enzymes to degrade fibrous proteins in the fibrous cap, leading to thinning of the plaque fibrous cap, transition of the plaque to an unstable state, and accelerated progression of atherosclerosis [33]. This contributes to poor short-term prognosis and indirectly supports the inference that the TyG index can reflect the degree of systemic inflammatory activation.

Research has shown that hyperglycemia directly caused by insulin resistance in the intensive care unit (including surgical intensive care units) can activate the immune system [34]. Similar mechanisms may exist in postoperative patients because postoperative changes in glucose metabolism are very similar to those in diabetes [35]. The affected organs are also almost identical (mainly non-insulin-dependent cells and organs), and the range of complications is very similar (including complications related to infection, kidney, cardiovascular system, and muscle weakness). However, unlike diabetic patients who typically take a long time to develop complications, complications in surgical patients progress more rapidly, often occurring within days or weeks. A study [36] found a significant relationship between insulin resistance and major complications, particularly infections. Their findings indicate that a 50% decrease in insulin sensitivity after surgery increases the risk of major complications by 5 to 6 times and increases the risk of severe infection by more than 10 times. The high incidence of infections in surgical patients caused by insulin resistance may prolong the recovery period of surgical intensive care patients [10]. Our research found that an elevated TyG index in critically ill patients in the surgical ICU is not only associated with the short-term prognosis of these patients but also affects their long-term prognosis. This suggests that postoperative patients may have long-term insulin resistance after surgery, and studies have found that this phenomenon is similar to the long-term insulin resistance seen in patients after severe burns [28, 37]. Long-term insulin resistance will adversely affect the long-term prognosis of chronic diseases such as stroke and coronary heart disease. This may also explain why an elevated TyG index after surgery is associated with long-term adverse outcomes in such patients.

Our study found differences in the distribution of TyG index and blood lipids between the elderly group (≥ 60) and the younger group (< 60). Younger individuals were more likely to have higher triglyceride levels and higher TyG index, as shown in Supplemental Table 1. Additionally, higher TyG was associated with prognosis in critically ill patients under 60 but not in patients over 60. The conclusions drawn align with those from the NHANES database [38], despite differences in our target population. The main reason for this age difference may be: (1) compared to younger patients, elderly patients have more comorbidities and poorer organ function, where the prognostic value of mortality in elderly patients may far outweigh that of the TyG index (with no additional prognostic value). (2) The use of antidiabetic drugs in elderly patients may lead to the TyG index not effectively reflecting the degree of insulin resistance. This also explains why we found similar conclusions in the diabetes subgroup. In non-diabetic patients, higher TyG index is associated with worse prognosis, as high TyG indicates higher insulin resistance and implies more severe trauma. However, in diabetic and elderly patients, the fluctuation of blood sugar due to the use of hypoglycemic drugs may prevent TyG from accurately reflecting the severity of trauma, thus rendering it unsuitable for prognosis evaluation. Many current studies suggest [39, 40] that triglyceride levels increase with age in the population before decreasing in middle age. In our study, we found significantly higher triglyceride levels in the younger group compared to the elderly group, and we speculate that this difference may be due to the use of lipid-lowering drugs.

Therefore, we believe that the TyG index has more diagnostic value for the surgical prognosis of nondiabetic patients. However, it should be noted that the TyG index in nondiabetic surgical patients only represents the severity of postoperative stress in patients, so symptomatic treatment for postoperative stress-induced hyperglycemia alone may not improve the prognosis of such patients. This suggests that comprehensive measures should be taken to reduce the stress response caused by surgical trauma. The introduction of “enhanced recovery after surgery” (ERAS) or “fast-track surgery” during the perioperative period is strong evidence in support of this conclusion. In the application of ERAS, as many treatment methods as possible are adopted to minimize surgical stress and accelerate functional recovery [41]. A recent meta-analysis [42] has shown that this perioperative management system is effective in reducing recovery time and stress-induced complications. In conclusion, adopting comprehensive measures to reduce the stress response caused by surgical trauma is a reasonable clinical decision to improve the surgical prognosis of these types of patients. Therefore, the TyG index may be used to dynamically assess the effectiveness of treatment, but this conclusion still needs further clinical research to confirm.

In our models, we attempted to use the acute physiology score III (APSIII) and sequential organ failure assessment (SOFA) combined with the TyG index to evaluate prognosis, analyzing the impact of the TyG index on the predictive ability of the scoring tools. Compared to the scoring tools without the TyG index, the predictive ability (C-index) of the scoring tools with the TyG index improved. However, this improvement was not statistically significant when assessed using integrated discriminant improvement (IDI) [43] and net reclassification improvement (NRI) [44] (*P* > 0.05)(Table S5). This may be because the SOFA score and APSIII score, as tools reflecting multiple organ functions, indicate the current severity of the patient’s condition and show some collinearity with the TyG index [45–47]. This could underestimate the significance of the TyG index in assessing critically ill surgical patients. However, there is currently no objective laboratory indicator to evaluate the patient’s stress state. The TyG index, as an indicator of post-traumatic insulin resistance, might potentially be used as a component in developing a new postoperative scoring system in the future.

Our study has certain limitations. Our results are derived from a population in American ICUs and may not be representative. Secondly, our study is an observational study, so it is impossible to completely avoid biases caused by confounding factors. Thirdly, only the initial TyG index upon ICU admission was used in our study, and the impact of this marker on prognosis over time could not be evaluated. Currently, the study [48] has shown that the variation index of the TyG index may have more diagnostic value. Fourth, the calculation of the TyG index can be influenced by medication. Finally, we included critically ill patients from all surgical ICUs and trauma ICUs, and when extrapolating to patients undergoing general surgery, the conclusions require more rigorous argumentation and larger RCT studies to confirm.

## Conclusion

An increasing risk of mortality was related to an elevated TyG index. In critically ill patients younger than 60 years old, as well as those with concomitant stroke or cardiovascular diseases, an elevated TyG index is associated with adverse short-term and long-term outcomes. Furthermore, in non-diabetic patients, an elevated TyG index is associated with adverse long-term prognosis.

### Electronic supplementary material

Below is the link to the electronic supplementary material.

Supplemental Fig. 1. Subgroup analyses for the association of TyG index(T1 vs. T2) with 28-day mortality. All model adjusted for age, sex, race/ethnicity, comorbidities except subgroup variable.HR, hazard ratio; CI, confidence interval; TyG, triglyceride–glucose.

Supplemental Fig. 2. Subgroup analyses for the association of TyG index(T1 vs. T3) with 28-day mortality. All model adjusted for age, sex, race/ethnicity, comorbidities except subgroup variable.HR, hazard ratio; CI, confidence interval; TyG, triglyceride–glucose.

Supplemental Fig. 3. Subgroup analyses for the association of TyG index(T1 vs. T2) with 180-day mortality. All model adjusted for age, sex, race/ethnicity, comorbidities except subgroup variable.HR, hazard ratio; CI, confidence interval; TyG, triglyceride–glucose.

Supplemental Fig. 4. Subgroup analyses for the association of TyG index(T1 vs. T3) with 180-day mortality. All model adjusted for age, sex, race/ethnicity, comorbidities except subgroup variable.HR, hazard ratio; CI, confidence interval; TyG, triglyceride–glucose.

Supplementary Material 5.

## Data Availability

The datasets generated and analyzed during the current study are available from the corresponding author on reasonable request.

## Abbreviations

APSIII: Acute physiology score III
CCS: Chronic coronary syndrome
CI: Confidence intervals
GCS: Glasgow Coma Scale
HbA1c: Glycated hemoglobin A1c
HR: Hazard ratio
ICD-10: International classification of diseases, tenth revision
ICU: Intensive care unit
IDI: Integrated discriminant improvement
INR: International normalized ratio
IQR: Interquartile range
IR: Insulin resistance
MACE: Major adverse cardiovascular events
MIMIC-IV: Medical Information Mart for Intensive Care IV
NO: Nitric oxide
NRI: Net reclassification improvement
OCT: Optical coherence tomography
PE: Plaque erosion
PR: Plaque rupture
PT: Prothrombin time
RAAS: Renin-angiotensin-aldosterone system
RCS: Restricted cubic spline
SD: Standard deviation
SICU: Surgical intensive care unit
SOFA: Sequential organ failure assessment score
SQL: Structured query language
STEMI: ST-elevation myocardial infarction
TSICU: Trauma surgical intensive care unit
TyG index: Triglyceride–glucose index
VAT: Visceral adipose tissue
ERAS: Enhanced recovery after surgery
