# Supplementary figures and images for: Association of the TyG index with prognosis in surgical intensive care patients: data from the MIMIC-IV
Source: Cardiovasc Diabetol. 2024 Jun 6;23:193. doi: 10.1186/s12933-024-02293-0 (PMC11157750; doi:10.1186/s12933-024-02293-0)

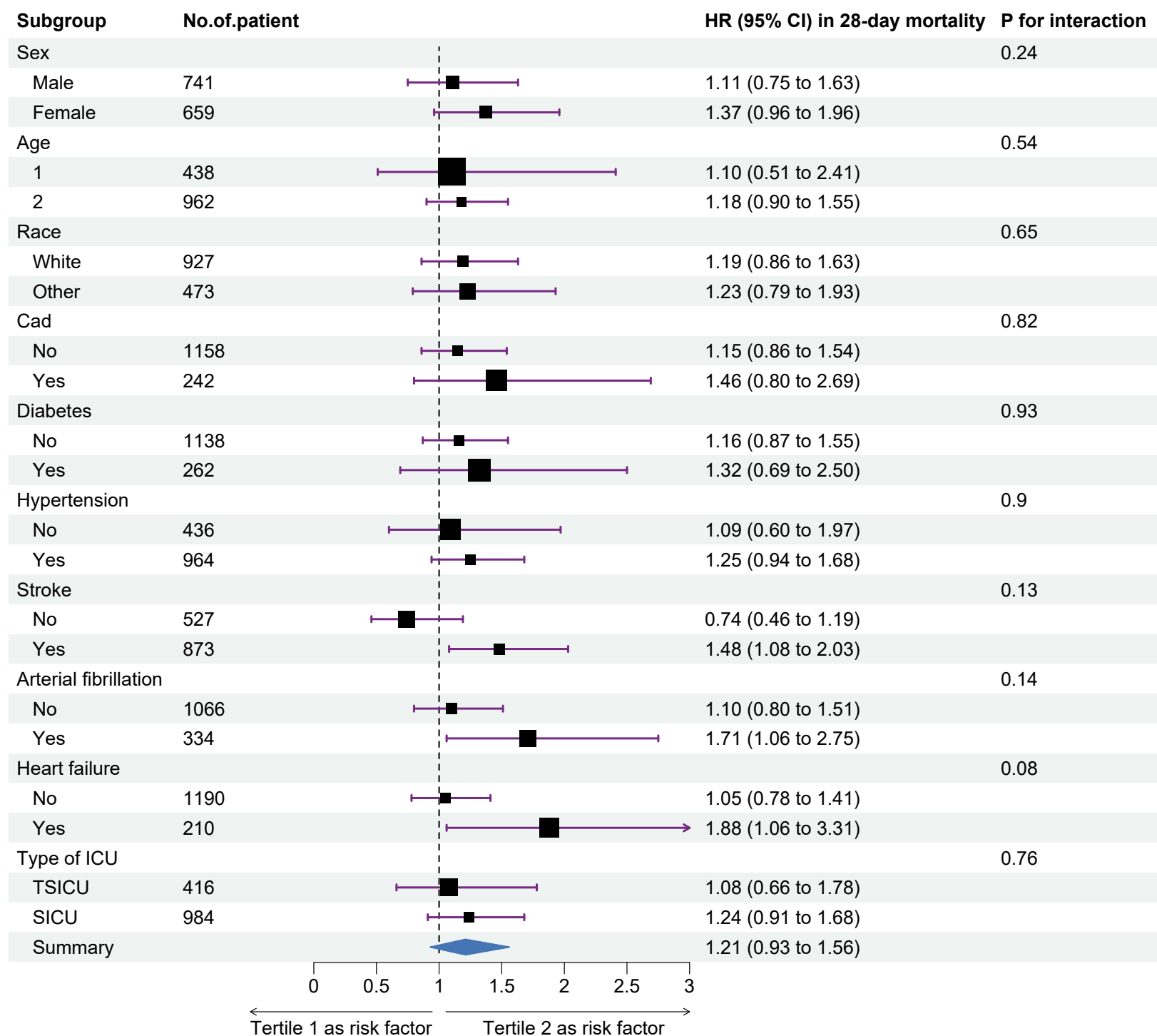

Supplement: Supplementary file 1 — Supplemental Fig. 1. Subgroup analyses for the association of TyG index(T1 vs. T2) with 28-day mortality. All model adjusted for age, sex, race/ethnicity, comorbidities except subgroup variable.HR, hazard ratio; CI, confidence interval; TyG, triglyceride–glucose. [file 12933_2024_2293_MOESM1_ESM.pdf]

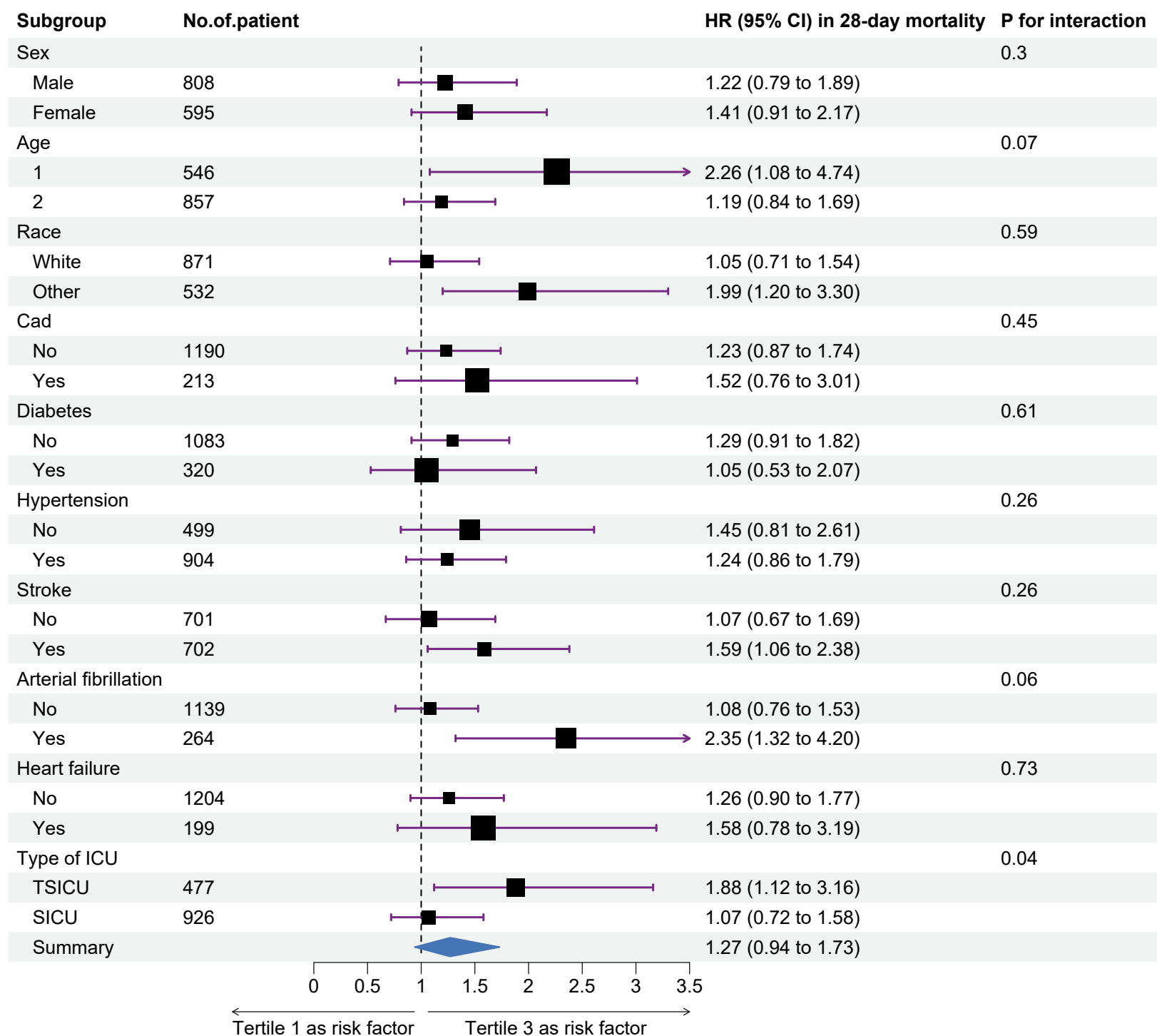

Supplement: Supplementary file 2 — Supplemental Fig. 2. Subgroup analyses for the association of TyG index(T1 vs. T3) with 28-day mortality. All model adjusted for age, sex, race/ethnicity, comorbidities except subgroup variable.HR, hazard ratio; CI, confidence interval; TyG, triglyceride–glucose. [file 12933_2024_2293_MOESM2_ESM.pdf]

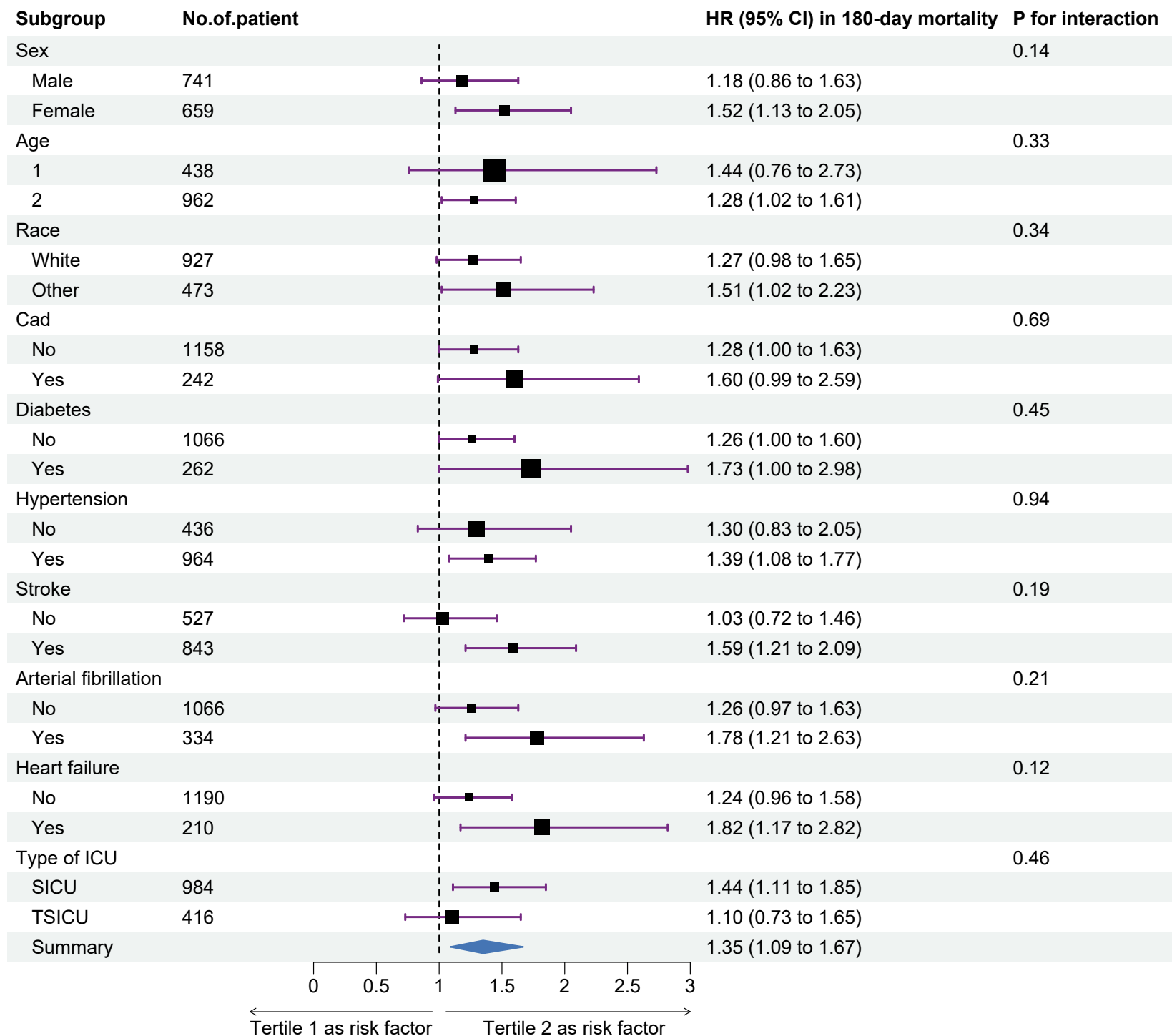

Supplement: Supplementary file 3 — Supplemental Fig. 3. Subgroup analyses for the association of TyG index(T1 vs. T2) with 180-day mortality. All model adjusted for age, sex, race/ethnicity, comorbidities except subgroup variable.HR, hazard ratio; CI, confidence interval; TyG, triglyceride–glucose. [file 12933_2024_2293_MOESM3_ESM.pdf]

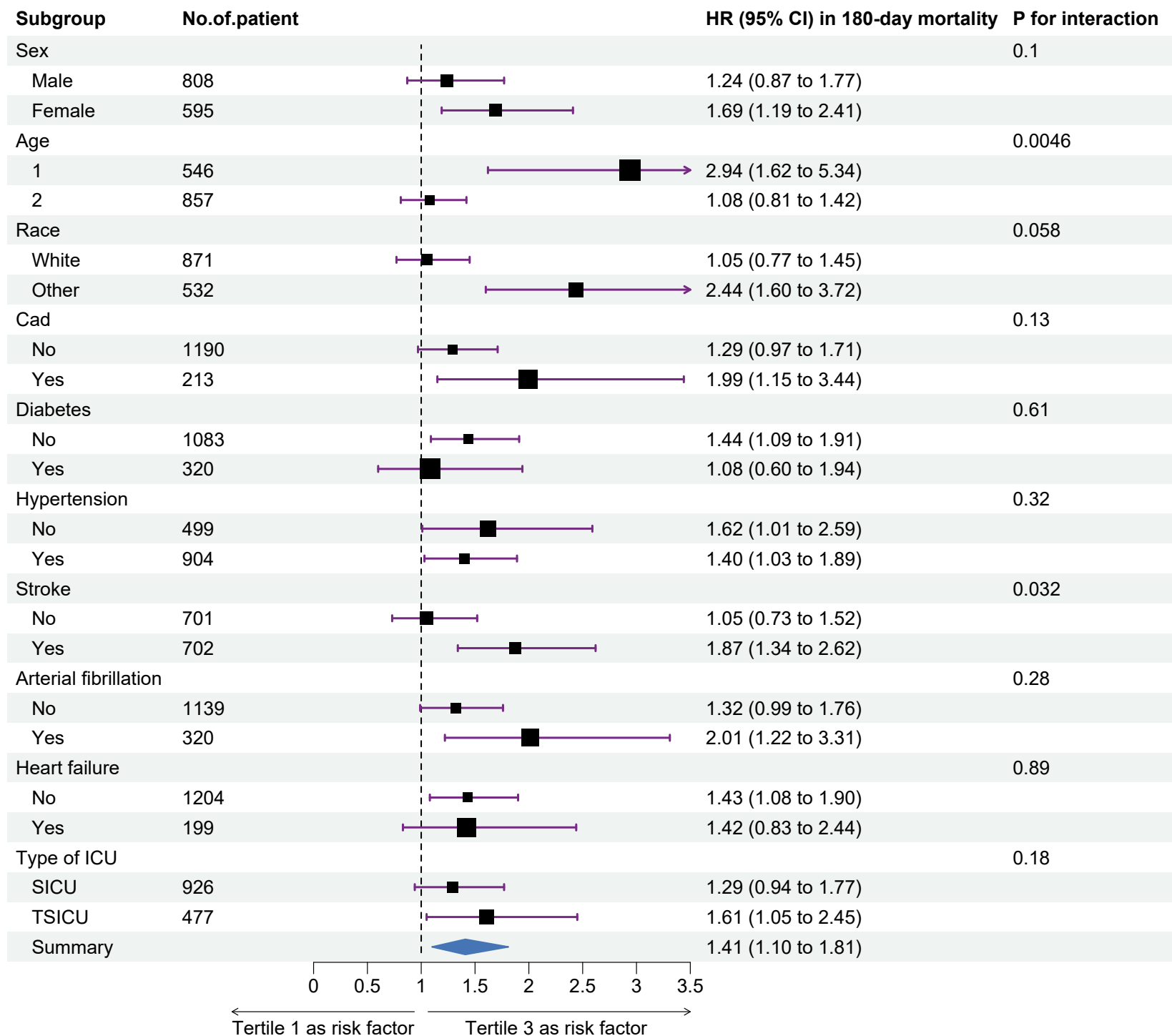

Supplement: Supplementary file 4 — Supplemental Fig. 4. Subgroup analyses for the association of TyG index(T1 vs. T3) with 180-day mortality. All model adjusted for age, sex, race/ethnicity, comorbidities except subgroup variable.HR, hazard ratio; CI, confidence interval; TyG, triglyceride–glucose. [file 12933_2024_2293_MOESM4_ESM.pdf]
