# Supplementary material for: Association of the TyG index with prognosis in surgical intensive care patients: data from the MIMIC-IV
Source: Cardiovasc Diabetol. 2024 Jun 6;23:193. doi: 10.1186/s12933-024-02293-0 (PMC11157750; doi:10.1186/s12933-024-02293-0)
Supplement: Supplementary file 5 — Supplementary Material 5. [file 12933_2024_2293_MOESM5_ESM.docx]

**Table S1. Characteristics and outcomes of participants categorized by age**

|  | **Age** | | |  | |
| --- | --- | --- | --- | --- | --- |
| **Characteristic** | **Overall**, N = 2,103^1^ | **<60**  N = 789^1^ | **≥ 60**  N = 1,314^1^ | **p-value**^2^ | **q-value**^3^ |
| **fasting blood glucose**  **(mg/dl)** | 166 (89) | 169 (102) | 165 (81) | 0.4 | >0.9 |
| **Triglyceride**  **(mg/dl)** | 180 (233) | 233 (300) | 148 (175) | **<0.001** | <0.001 |
| **TyG Index** | 9.20 (0.84) | 9.40 (0.93) | 9.08 (0.76) | **<0.001** | <0.001 |
| **TyG group** |  |  |  | **<0.001** | <0.001 |
| T1 | 700 (33%) | 195 (25%) | 505 (38%) |  |  |
| T2 | 700 (33%) | 243 (31%) | 457 (35%) |  |  |
| T3 | 703 (33%) | 351 (44%) | 352 (27%) |  |  |
| **28-day**  **Mortality** | 375 (18%) | 82 (10%) | 293 (22%) | **<0.001** | <0.001 |
| **180-day**  **Mortality** | 559 (27%) | 129 (16%) | 430 (33%) | **<0.001** | <0.001 |
| ^1^Mean (SD); n (%) | | | | | |
| ^2^Wilcoxon rank sum test; Pearson's Chi-squared test | | | | | |
| ^3^Bonferroni correction for multiple testing | | | | | |

**Table S2. Characteristics and outcomes of participants categorized by Diabetes**

|  | **Diabetes** | | |  | |
| --- | --- | --- | --- | --- | --- |
| **Characteristic** | **Overall**, N = 2,103^1^ | **No**  , N = 1,608^1^ | **Yes**  , N = 495^1^ | **p-value**^2^ | **q-value**^3^ |
| **age** | 64 (17) | 63 (18) | 68 (14) | **<0.001** | <0.001 |
| **fasting blood glucose**  **(mg/dl)** | 166 (89) | 153 (81) | 209 (101) | **<0.001** | <0.001 |
| **Triglyceride**  **(mg/dl)** | 180 (233) | 177 (236) | 190 (223) | **<0.001** | 0.003 |
| **TyG Index** | 9.20 (0.84) | 9.10 (0.82) | 9.50 (0.85) | **<0.001** | <0.001 |
| **TyG group** |  |  |  | **<0.001** | <0.001 |
| T1 | 700 (33%) | 613 (38%) | 87 (18%) |  |  |
| T2 | 700 (33%) | 525 (33%) | 175 (35%) |  |  |
| T3 | 703 (33%) | 470 (29%) | 233 (47%) |  |  |
| **28-day**  **Mortality** | 375 (18%) | 287 (18%) | 88 (18%) | >0.9 | >0.9 |
| **180-day**  **Mortality** | 559 (27%) | 431 (27%) | 128 (26%) | 0.7 | >0.9 |
| ^1^Mean (SD); n (%) | | | | | |
| ^2^Wilcoxon rank sum test; Pearson's Chi-squared test | | | | | |
| ^3^Bonferroni correction for multiple testing | | | | | |

**Table S3. Characteristics and outcomes of participants categorized by Stroke**

|  | **Stroke** | | |  | |
| --- | --- | --- | --- | --- | --- |
| **Characteristic** | **Overall**, N = 2,103^1^ | **No**  , N = 979^1^ | **Yes**  , N = 1,124^1^ | **p-value**^2^ | **q-value**^3^ |
| **age** | 64 (17) | 58 (18) | 69 (16) | **<0.001** | <0.001 |
| **fasting blood glucose**  **(mg/dl)** | 166 (89) | 181 (103) | 154 (73) | **<0.001** | <0.001 |
| **Triglyceride**  **(mg/dl)** | 180 (233) | 227 (302) | 138 (138) | **<0.001** | <0.001 |
| **TyG Index** | 9.20 (0.84) | 9.43 (0.93) | 8.99 (0.69) | **<0.001** | <0.001 |
| **TyG group** |  |  |  | **<0.001** | <0.001 |
| T1 | 700 (33%) | 249 (25%) | 451 (40%) |  |  |
| T2 | 700 (33%) | 278 (28%) | 422 (38%) |  |  |
| T3 | 703 (33%) | 452 (46%) | 251 (22%) |  |  |
| **28-day**  **Mortality** | 375 (18%) | 144 (15%) | 231 (21%) | **<0.001** | 0.003 |
| **180-day**  **Mortality** | 559 (27%) | 243 (25%) | 316 (28%) | 0.088 | 0.6 |
| ^1^Mean (SD); n (%) | | | | | |
| ^2^Wilcoxon rank sum test; Pearson's Chi-squared test | | | | | |
| ^3^Bonferroni correction for multiple testing | | | | | |

**Table S4. Characteristics and outcomes of participants categorized by cad**

|  | **CAD** | | |  | |
| --- | --- | --- | --- | --- | --- |
| **Characteristic** | **Overall**, N = 2,103^1^ | **No**,  N = 1,762^1^ | **Yes**  N = 341^1^ | **p-value**^2^ | **q-value**^3^ |
| **age** | 64 (17) | 62 (18) | 73 (12) | **<0.001** | <0.001 |
| **fasting blood glucose**  **(mg/dl)** | 166 (89) | 166 (92) | 171 (75) | **0.014** | 0.095 |
| **Triglyceride**  **(mg/dl)** | 180 (233) | 184 (241) | 157 (191) | **0.014** | 0.10 |
| **TyG Index** | 9.20 (0.84) | 9.21 (0.85) | 9.14 (0.83) | 0.4 | >0.9 |
| **TyG group** |  |  |  | 0.10 | 0.7 |
| T1 | 700 (33%) | 586 (33%) | 114 (33%) |  |  |
| T2 | 700 (33%) | 572 (32%) | 128 (38%) |  |  |
| T3 | 703 (33%) | 604 (34%) | 99 (29%) |  |  |
| **28-day**  **Mortality** | 375 (18%) | 299 (17%) | 76 (22%) | **0.019** | 0.13 |
| **180-day**  **Mortality** | 559 (27%) | 435 (25%) | 124 (36%) | **<0.001** | <0.001 |
| ^1^Mean (SD); n (%) | | | | | |
| ^2^Wilcoxon rank sum test; Pearson's Chi-squared test | | | | | |
| ^3^Bonferroni correction for multiple testing | | | | | |

**Table S5. The incremental effect of the TyG index**

| Models | C-index | IDI (95%CI)  P-value | NRI (95%CI)  P-value |
| --- | --- | --- | --- |
| 28-day mortality |  |  |  |
| SOFA | 0.704 | 0.003(-0.001,0.019)  0.240 | 0.072(-0.072,0.199)  0.271 |
| SOFA+TyG | 0.716 |  |  |
| APSIII | 0.746 | 0.002(-0.002,0.016)  0.395 | 0.029(-0.196,0.157)  0.487 |
| APSIII+TyG | 0.752 |  |  |
| 180-day mortality |  |  |  |
| SOFA | 0.681 | 0.006(-0.002,0.025)  0.188 | 0.136(0.008,0.238)  0.056 |
| SOFA+TyG | 0.707 |  |  |
| APSIII | 0.732 | 0.003(-0.003,0.017)  0.407 | 0.083(-0.175,0.189)  0.192 |
| APSIII+TyG | 0.745 |  |  |

IDI: integrated discriminant improvement; NRI: net reclassification improvement

**Table S6. VIF in Table 2**

| VIF | Model in table 2 | TyG(as number) | TyG(as category ) | ICU type | Age | Race | Sex | Weight | eGFR | HF | HTN | AF | DM | Renal | Liver | COPD | Cad | Stroke | Tumor |
| --- | --- | --- | --- | --- | --- | --- | --- | --- | --- | --- | --- | --- | --- | --- | --- | --- | --- | --- | --- |
| 28 day | Model 2 | 1.10 |  |  | 1.17 | 1.02 | 1.05 |  |  |  |  |  |  |  |  |  |  |  |  |
|  | Model 2 |  | 1.02 |  | 1.07 | 1.01 | 1.02 |  |  |  |  |  |  |  |  |  |  |  |  |
|  | Model 3 | 1.35 |  | 1.06 | 1.83 | 1.05 | 1.20 | 1.36 | 1.56 | 1.21 | 1.25 | 1.16 | 1.16 | 1.50 | 1.21 | 1.04 | 1.14 | 1.36 | 1.03 |
|  | Model 3 |  | 1.06 | 1.03 | 1.35 | 1.03 | 1.10 | 1.15 | 1.24 | 1.10 | 1.12 | 1.08 | 1.08 | 1.23 | 1.098 | 1.02 | 1.06 | 1.15 | 1.01 |
| 180day | Model 2 | 1.10 |  |  | 1.17 | 1.03 | 1.05 |  |  |  |  |  |  |  |  |  |  |  |  |
|  | Model 2 |  | 1.02 |  | 1.08 | 1.01 | 1.03 |  |  |  |  |  |  |  |  |  |  |  |  |
|  | Model 3 | 1.34 |  | 1.06 | 1.83 | 1.05 | 1.21 | 1.34 | 1.57 | 1.22 | 1.27 | 1.16 | 1.17 | 1.51 | 1.17 | 1.04 | 1.15 | 1.35 | 1.04 |
|  | Model 3 |  | 1.06 | 1.03 | 1.35 | 1.03 | 1.10 | 1.15 | 1.25 | 1.10 | 1.13 | 1.08 | 1.07 | 1.50 | 1.08 | 1.02 | 1.07 | 1.15 | 1.25 |

HF, heart failure; HTN, hypertension; AF, arterial fibrillation; DM, diabetes; Renal, renal disease; Liver, liver disease; cad, cardiovascular disease;

| VIF | Model in table 2 | TyG(as number) | TyG(as category ) | ICU type | Age | Race | Sex | Weight | eGFR | HF | HTN | AF | DM | Renal | Liver | COPD | Cad | Stroke | Tumor |
| --- | --- | --- | --- | --- | --- | --- | --- | --- | --- | --- | --- | --- | --- | --- | --- | --- | --- | --- | --- |
| 28 day | Model 2 | 1.10 |  |  | 1.17 | 1.02 | 1.05 |  |  |  |  |  |  |  |  |  |  |  |  |
|  | Model 2 |  | 1.02 |  | 1.07 | 1.01 | 1.02 |  |  |  |  |  |  |  |  |  |  |  |  |
|  | Model 3 | 1.35 |  | 1.06 | 1.83 | 1.05 | 1.20 | 1.36 | 1.56 | 1.21 | 1.25 | 1.16 | 1.16 | 1.50 | 1.21 | 1.04 | 1.14 | 1.36 | 1.03 |
|  | Model 3 |  | 1.06 | 1.03 | 1.35 | 1.03 | 1.10 | 1.15 | 1.24 | 1.10 | 1.12 | 1.08 | 1.08 | 1.23 | 1.098 | 1.02 | 1.06 | 1.15 | 1.01 |
| 180day | Model 2 | 1.10 |  |  | 1.17 | 1.03 | 1.05 |  |  |  |  |  |  |  |  |  |  |  |  |
|  | Model 2 |  | 1.02 |  | 1.08 | 1.01 | 1.03 |  |  |  |  |  |  |  |  |  |  |  |  |
|  | Model 3 | 1.34 |  | 1.06 | 1.83 | 1.05 | 1.21 | 1.34 | 1.57 | 1.22 | 1.27 | 1.16 | 1.17 | 1.51 | 1.17 | 1.04 | 1.15 | 1.35 | 1.04 |
|  | Model 3 |  | 1.06 | 1.03 | 1.35 | 1.03 | 1.10 | 1.15 | 1.25 | 1.10 | 1.13 | 1.08 | 1.07 | 1.50 | 1.08 | 1.02 | 1.07 | 1.15 | 1.25 |

**Table S7. VIF in Supplemental Figure 1 (T1 vs T2 in 28-day mortality)**

| subgroup |  | TyG(as category ) | ICU type | Age | Race | Sex | Weight | eGFR | HF | HTN | AF | DM | Renal | Liver | COPD | Cad | Stroke | Tumor |
| --- | --- | --- | --- | --- | --- | --- | --- | --- | --- | --- | --- | --- | --- | --- | --- | --- | --- | --- |
| Sex | Male | 1.19 | 1.05 | 1.56 | 1.07 |  | 1.17 | 1.19 | 1.21 | 1.19 | 1.15 | 1.10 | 1.61 | 1.15 | 1.19 | 1.20 | 1.28 | 1.06 |
| 1.65 | Female | 1.05 | 1.05 | 1.77 | 1.07 |  | 1.19 | 1.63 | 1.23 | 1.25 | 1.14 | 1.15 | 1.56 | 1.38 | 1.03 | 1.16 | 1.29 | 1.03 |
| Age | <60 | 1.17 | 1.16 |  | 1.20 | 1.25 | 1.15 | 1.43 | 1.12 | 1.32 | 1.29 | 1.36 | 1.37 | 1.66 | 1.36 | 1.10 | 1.62 | 1.05 |
|  | ≥60 | 1.07 | 1.02 |  | 1.05 | 1.22 | 1.21 | 1.59 | 1.19 | 1.05 | 1.08 | 1.10 | 1.65 | 1.08 | 1.05 | 1.13 | 1.16 | 1.02 |
| Race | White | 1.09 | 1.03 | 1.76 |  | 1.21 | 1.37 | 1.49 | 1.21 | 1.28 | 1.14 | 1.10 | 1.46 | 1.30 | 1.08 | 1.17 | 1.27 | 1.01 |
|  | Other | 1.15 | 1.07 | 1.67 |  | 1.16 | 1.30 | 1.88 | 1.22 | 1.16 | 1.16 | 1.16 | 1.77 | 1.15 | 1.13 | 1.15 | 1.25 | 1.14 |
| CAD | No | 1.11 | 1.05 | 1.76 | 1.06 | 1.17 | 1.28 | 1.57 | 1.14 | 1.24 | 1.16 | 1.09 | 1.41 | 1.27 | 1.02 |  | 1.36 | 1.03 |
|  | Yes | 1.24 | 1.07 | 1.42 | 1.16 | 1.37 | 1.48 | 1.82 | 1.14 | 1.19 | 1.15 | 1.15 | 1.82 | 1.18 | 1.15 |  | 1.13 | 1.07 |
| Diabetes | No | 1.09 | 1.03 | 1.77 | 1.06 | 1.22 | 1.29 | 1.52 | 1.21 | 1.21 | 1.16 |  | 1.45 | 1.24 | 1.07 | 1.15 | 1.26 | 1.03 |
|  | Yes | 1.12 | 1.10 | 1.52 | 1.15 | 1.21 | 1.41 | 1.98 | 1.26 | 1.13 | 1.14 |  | 1.95 | 1.21 | 1.17 | 1.17 | 1.42 | 1.11 |
| Hypertension | No | 1.21 | 1.11 | 1.70 | 1.15 | 1.18 | 1.31 | 1.44 | 1.44 |  | 1.38 | 1.20 | 1.28 | 1.67 | 1.24 | 1.23 | 1.63 | 1.18 |
|  | Yes | 1.10 | 1.02 | 1.49 | 1.09 | 1.22 | 1.37 | 1.66 | 1.17 |  | 1.10 | 1.08 | 1.61 | 1.12 | 1.04 | 1.13 | 1.14 | 1.03 |
| Stroke | No | 1.13 | 1.05 | 1.79 | 1.03 | 1.11 | 1.21 | 1.43 | 1.36 | 1.38 | 1.13 | 1.15 | 1.47 | 1.30 | 1.07 | 1.14 |  | 1.07 |
|  | Yes | 1.15 | 1.02 | 1.51 | 1.10 | 1.36 | 1.44 | 1.77 | 1.20 | 1.08 | 1.11 | 1.10 | 1.66 | 1.12 | 1.09 | 1.16 |  | 1.04 |
| AF | No | 1.09 | 1.05 | 1.67 | 1.08 | 1.16 | 1.30 | 1.64 | 1.22 | 1.29 |  | 1.12 | 1.53 | 1.25 | 1.06 | 1.15 | 1.30 | 1.03 |
|  | Yes | 1.19 | 1.03 | 1.48 | 1.08 | 1.30 | 1.38 | 1.71 | 1.14 | 1.15 |  | 1.15 | 1.77 | 1.25 | 1.11 | 1.22 | 1.19 | 1.17 |
| Heart failure | No | 1.09 | 1.04 | 1.72 | 1.05 | 1.19 | 1.34 | 1.50 |  | 1.21 | 1.10 | 1.08 | 1.33 | 1.31 | 1.044 | 1.077 | 1.37 | 1.03 |
|  | Yes | 1.27 | 1.05 | 1.44 | 1.24 | 1.35 | 1.42 | 1.63 |  | 1.33 | 1.21 | 1.26 | 1.83 | 1.31 | 1.20 | 1.20 | 1.27 | 1.14 |
| Type of ICU | TSICU | 1.10 |  | 1.66 | 1.08 | 1.20 | 1.33 | 1.65 | 1.20 | 1.18 | 1.12 | 1.08 | 1.57 | 1.21 | 1.07 | 1.15 | 1.25 | 1.03 |
|  | SICU | 1.10 |  | 1.75 | 1.05 | 1.25 | 1.30 | 1.49 | 1.35 | 1.36 | 1.28 | 1.18 | 1.61 | 1.18 | 1.11 | 1.20 | 1.25 | 1.11 |

**Table S8. VIF in Supplemental Figure 2 (T1 vs T3 in 28-day mortality)**

| subgroup |  | TyG(as category ) | ICU type | Age | Race | Sex | Weight | eGFR | HF | HTN | AF | DM | Renal | Liver | COPD | Cad | Stroke | Tumor |
| --- | --- | --- | --- | --- | --- | --- | --- | --- | --- | --- | --- | --- | --- | --- | --- | --- | --- | --- |
| Sex | Male | 1.62 | 1.11 | 1.85 | 1.11 |  | 1.29 | 1.67 | 1.25 | 1.27 | 1.15 | 1.24 | 1.63 | 1.19 | 1.06 | 1.21 | 1.43 | 1.06 |
|  | Female | 1.35 | 1.09 | 1.84 | 1.05 |  | 1.20 | 1.43 | 1.26 | 1.42 | 1.23 | 1.18 | 1.44 | 1.23 | 1.03 | 1.10 | 1.45 | 1.03 |
| Age | <60 | 1.26 | 1.13 |  | 1.04 | 1.15 | 1.31 | 1.50 | 1.09 | 1.38 | 1.07 | 1.36 | 1.42 | 1.29 | 1.23 | 1.14 | 1.28 | 1.11 |
|  | ≥60 | 1.31 | 1.06 |  | 1.05 | 1.20 | 1.26 | 1.58 | 1.24 | 1.13 | 1.08 | 1.21 | 1.65 | 1.16 | 1.03 | 1.12 | 1.30 | 1.04 |
| Race | White | 1.41 | 1.05 | 1.82 |  | 1.22 | 1.43 | 1.54 | 1.17 | 1.33 | 1.16 | 1.20 | 1.46 | 1.23 | 1.04 | 1.15 | 1.32 | 1.02 |
|  | Other | 1.53 | 1.17 | 1.92 |  | 1.20 | 1.35 | 1.66 | 1.43 | 1.33 | 1.26 | 1.31 | 1.74 | 1.30 | 1.14 | 1.23 | 1.49 | 1.08 |
| CAD | No | 1.48 | 1.09 | 1.92 | 1.07 | 1.17 | 1.37 | 1.54 | 1.18 | 1.33 | 1.20 | 1.19 | 1.41 | 1.22 | 1.03 |  | 1.47 | 1.04 |
|  | Yes | 1.46 | 1.16 | 1.62 | 1.15 | 1.31 | 1.54 | 1.93 | 1.22 | 1.14 | 1.17 | 1.40 | 2.22 | 1.30 | 1.08 |  | 1.25 | 1.15 |
| Diabetes | No | 1.42 | 1.08 | 2.05 | 1.05 | 1.17 | 1.32 | 1.51 | 1.27 | 1.31 | 1.19 |  | 1.43 | 1.21 | 1.03 | 1.12 | 1.44 | 1.02 |
|  | Yes | 1.29 | 1.11 | 1.57 | 1.19 | 1.27 | 1.49 | 1.75 | 1.26 | 1.31 | 1.07 |  | 1.81 | 1.42 | 1.16 | 1.32 | 1.40 | 1.19 |
| Hypertension | No | 1.47 | 1.20 | 1.61 | 1.07 | 1.24 | 1.32 | 1.54 | 1.37 |  | 1.41 | 1.18 | 1.39 | 1.39 | 1.16 | 1.22 | 1.46 | 1.04 |
|  | Yes | 1.46 | 1.06 | 1.69 | 1.08 | 1.19 | 1.43 | 1.56 | 1.20 |  | 1.12 | 1.19 | 1.55 | 1.15 | 1.01 | 1.10 | 1.25 | 1.02 |
| Stroke | No | 1.32 | 1.06 | 1.72 | 1.06 | 1.16 | 1.33 | 1.35 | 1.27 | 1.32 | 1.12 | 1.17 | 1.38 | 1.18 | 1.09 | 1.18 |  | 1.04 |
|  | Yes | 1.37 | 1.04 | 1.71 | 1.09 | 1.25 | 1.40 | 1.80 | 1.27 | 1.13 | 1.14 | 1.30 | 1.70 | 1.09 | 1.04 | 1.15 |  | 1.03 |
| AF | No | 1.43 | 1.08 | 1.76 | 1.05 | 1.17 | 1.35 | 1.53 | 1.20 | 1.35 |  | 1.23 | 1.46 | 1.21 | 1.04 | 1.18 | 1.36 | 1.02 |
|  | Yes | 1.38 | 1.14 | 1.68 | 1.17 | 1.34 | 1.56 | 2.12 | 1.25 | 1.21 |  | 1.23 | 2.21 | 1.33 | 1.06 | 1.21 | 1.32 | 1.14 |
| Heart failure | No | 1.45 | 1.10 | 1.89 | 1.07 | 1.18 | 1.35 | 1.45 |  | 1.32 | 1.14 | 1.18 | 1.29 | 1.29 | 1.04 | 1.09 | 1.47 | 1.02 |
|  | Yes | 1.56 | 1.16 | 1.55 | 1.10 | 1.26 | 1.69 | 1.84 |  | 1.41 | 1.18 | 1.38 | 1.90 | 1.22 | 1.08 | 1.11 | 1.42 | 1.09 |
| Type of ICU | TSICU | 1.49 |  | 1.74 | 1.08 | 1.18 | 1.35 | 1.62 | 1.23 | 1.23 | 1.16 | 1.24 | 1.64 | 1.26 | 1.06 | 1.16 | 1.35 | 1.03 |
|  | SICU | 1.41 |  | 2.08 | 1.07 | 1.23 | 1.48 | 1.62 | 1.34 | 1.50 | 1.18 | 1.17 | 1.45 | 1.24 | 1.13 | 1.19 | 1.31 | 1.03 |

**Table S9. VIF in Supplemental Figure 3 (T1 vs T2 in 180-day mortality)**

| subgroup |  | TyG(as category ) | ICU type | Age | Race | Sex | Weight | eGFR | HF | HTN | AF | DM | Renal | Liver | COPD | Cad | Stroke | Tumor |
| --- | --- | --- | --- | --- | --- | --- | --- | --- | --- | --- | --- | --- | --- | --- | --- | --- | --- | --- |
| Sex | Male | 1.19 | 1.05 | 1.60 | 1.08 |  | 1.11 | 1.69 | 1.22 | 1.20 | 1.15 | 1.10 | 1.66 | 1.14 | 1.20 | 1.21 | 1.31 | 1.08 |
|  | Female | 1.06 | 1.05 | 1.74 | 1.07 |  | 1.21 | 1.64 | 1.24 | 1.21 | 1.15 | 1.15 | 1.55 | 1.28 | 1.03 | 1.18 | 1.25 | 1.03 |
| Age | <60 | 1.17 | 1.14 |  | 1.14 | 1.25 | 1.13 | 1.44 | 1.14 | 1.26 | 1.23 | 1.15 | 1.34 | 1.43 | 1.34 | 1.10 | 1.52 | 1.17 |
|  | ≥60 | 1.07 | 1.02 |  | 1.05 | 1.22 | 1.21 | 1.59 | 1.19 | 1.07 | 1.08 | 1.11 | 1.64 | 1.07 | 1.05 | 1.14 | 1.19 | 1.04 |
| Race | White | 1.09 | 1.03 | 1.78 |  | 1.22 | 1.34 | 1.57 | 1.23 | 1.31 | 1.15 | 1.11 | 1.51 | 1.25 | 1.10 | 1.21 | 1.25 | 1.03 |
|  | Other | 1.16 | 1.06 | 1.67 |  | 1.17 | 1.28 | 1.85 | 1.19 | 1.19 | 1.19 | 1.16 | 1.73 | 1.15 | 1.13 | 1.15 | 1.26 | 1.19 |
| CAD | No | 1.11 | 1.05 | 1.79 | 1.06 | 1.18 | 1.27 | 1.57 | 1.15 | 1.26 | 1.18 | 1.08 | 1.39 | 1.23 | 1.02 |  | 1.35 | 1.05 |
|  | Yes | 1.23 | 1.06 | 1.39 | 1.19 | 1.38 | 1.43 | 1.78 | 1.15 | 1.27 | 1.16 | 1.16 | 1.91 | 1.27 | 1.18 |  | 1.12 | 1.07 |
| Diabetes | No | 1.09 | 1.02 | 1.80 | 1.06 | 1.24 | 1.30 | 1.55 | 1.21 | 1.21 | 1.16 |  | 1.42 | 1.22 | 1.07 | 1.13 | 1.26 | 1.04 |
|  | Yes | 1.09 | 1.11 | 1.47 | 1.14 | 1.21 | 1.33 | 2.10 | 1.27 | 1.15 | 1.19 |  | 2.2 | 1.12 | 1.19 | 1.18 | 1.47 | 1.15 |
| Hypertension | No | 1.22 | 1.12 | 1.63 | 1.12 | 1.19 | 1.27 | 1.49 | 1.48 |  | 1.44 | 1.17 | 1.41 | 1.45 | 1.21 | 1.28 | 1.53 | 1.27 |
|  | Yes | 1.09 | 1.02 | 1.49 | 1.09 | 1.22 | 1.36 | 1.63 | 1.16 |  | 1.10 | 1.08 | 1.60 | 1.13 | 1.04 | 1.15 | 1.15 | 1.03 |
| Stroke | No | 1.12 | 1.04 | 1.76 | 1.04 | 1.12 | 1.21 | 1.55 | 1.34 | 1.36 | 1.15 | 1.14 | 1.52 | 1.21 | 1.11 | 1.18 |  | 1.07 |
|  | Yes | 1.15 | 1.03 | 1.51 | 1.11 | 1.36 | 1.43 | 1.73 | 1.21 | 1.08 | 1.11 | 1.11 | 1.62 | 1.11 | 1.09 | 1.16 |  | 1.06 |
| AF | No | 1.09 | 1.03 | 1.67 | 1.07 | 1.16 | 1.28 | 1.61 | 1.24 | 1.32 |  | 1.12 | 1.52 | 1.20 | 1.07 | 1.15 | 1.29 | 1.05 |
|  | Yes | 1.18 | 1.04 | 1.45 | 1.10 | 1.32 | 1.39 | 1.79 | 1.16 | 1.11 |  |  | 1.16 | 1.81 | 1.11 | 1.22 | 1.18 | 1.20 |
| Heart failure | No | 1.09 | 1.05 | 1.71 | 1.05 | 1.19 | 1.32 | 1.511 |  | 1.22 | 1.12 | 1.08 | 1.37 | 1.27 | 1.05 | 1.07 | 1.37 | 1.04 |
|  | Yes | 1.19 | 1.07 | 1.47 | 1.22 | 1.35 | 1.41 | 1.72 |  | 1.29 | 1.23 | 1.20 | 1.84 | 1.24 | 1.19 | 1.17 | 1.25 | 1.17 |
| Type of ICU | TSICU | 1.10 |  | 1.74 | 1.07 | 1.21 | 1.32 | 1.68 | 1.24 | 1.23 | 1.14 | 1.08 | 1.58 | 1.20 | 1.08 | 1.17 | 1.27 | 1.05 |
|  | SICU | 1.13 |  | 1.67 | 1.06 | 1.26 | 1.28 | 1.57 | 1.34 | 1.30 | 1.26 | 1.21 | 1.62 | 1.22 | 1.10 | 1.20 | 1.24 | 1.15 |

**Table S10. VIF in Supplemental Figure 4(T1 vs T3 in 180-day mortality)**

| subgroup |  | TyG(as category ) | ICU type | Age | Race | Sex | Weight | eGFR | HF | HTN | AF | DM | Renal | Liver | COPD | Cad | Stroke | Tumor |
| --- | --- | --- | --- | --- | --- | --- | --- | --- | --- | --- | --- | --- | --- | --- | --- | --- | --- | --- |
| Sex | Male | 1.56 | 1.18 | 1.86 | 1.10 |  | 1.25 | 1.73 | 1.23 | 1.28 | 1.14 | 1.23 | 1.67 | 1.14 | 1.09 | 1.21 | .141 | 1.08 |
|  | Female | 1.33 | 1.10 | 1.93 | 1.05 |  | 1.22 | 1.46 | 1.30 | 1.41 | 1.22 | 1.19 | 1.48 | 1.22 | 1.03 | 1.12 | 1.42 | 1.05 |
| Age | <60 | 1.22 | 1.14 |  | 1.03 | 1.13 | 1.28 | 1.42 | 1.13 | 1.35 | 1.09 | 1.34 | 1.41 | 1.25 | 1.20 | 1.11 | 1.30 | 1.12 |
|  | ≥60 | 1.28 | 1.06 |  | 1.05 | 1.20 | 1.27 | 1.60 | 1.23 | 1.14 | 1.08 | 1.20 | 1.69 | 1.12 | 1.04 | 1.12 | 1.26 | 1.04 |
| Race | White | 1.41 | 1.05 | 1.81 |  | 1.22 | 1.41 | 1.58 | 1.17 | 1.35 | 1.16 | 1.21 | 1.49 | 1.19 | 1.04 | 1.16 | 1.30 | 1.02 |
|  | Other | 1.47 | 1.17 | 1.99 |  | 1.20 | 1.33 | 1.71 | 1.47 | 1.38 | 1.25 | 1.31 | 1.78 | 1.23 | 1.14 | 1.21 | 1.50 | 1.13 |
| CAD | No | 1.46 | 1.10 | 1.92 | 1.06 | 1.16 | 1.35 | 1.57 | 1.18 | 1.35 | 1.22 | 1.18 | 1.44 | 1.17 | 1.04 |  | 1.47 | 1.06 |
|  | Yes | 1.49 | 1.16 | 1.68 | 1.14 | 1.35 | 1.56 | 1.83 | 1.17 | 1.15 | 1.15 | 1.39 | 2.11 | 1.33 | 1.11 |  | 1.21 | 1.14 |
| Diabetes | No | 1.4 | 1.08 | 2.02 | 1.05 | 1.18 | 1.31 | 1.53 | 1.25 | 1.31 | 1.20 |  | 1.43 | 1.17 | 1.05 | 1.12 | 1.42 | 1.03 |
|  | Yes | 1.23 | 1.12 | 1.57 | 1.14 | 1.24 | 1.51 | 1.72 | 1.24 | 1.28 | 1.06 |  | 1.80 | 1.28 | 1.16 | 1.36 | 1.35 | 1.12 |
| Hypertension | No | 1.50 | 1.19 | 1.64 | 1.05 | 1.24 | 1.33 | 1.53 | 1.37 |  | 1.43 | 1.14 | 1.42 | 1.34 | 1.17 | 1.24 | 1.48 | 1.07 |
|  | Yes | 1.44 | 1.06 | 1.69 | 1.08 | 1.18 | 1.43 | 1.58 | 1.20 |  | 1.12 | 1.18 | 1.59 | 1.13 | 1.02 | 1.11 | 1.23 | 1.03 |
| Stroke | No | 1.33 | 1.07 | 1.78 | 1.06 | 1.13 | 1.32 | 1.44 | 1.25 | 1.34 | 1.14 | 1.20 | 1.42 | 1.13 | 1.08 | 1.20 |  | 1.03 |
|  | Yes | 1.35 | 1.06 | 1.72 | 1.08 | 1.25 | 1.39 | 1.84 | 1.32 | 1.16 | 1.15 | 1.29 | 1.76 | 1.15 | 1.05 | 1.16 |  | 1.05 |
| AF | No | 1.40 | 1.08 | 1.76 | 1.04 | 1.16 | 1.33 | 1.53 | 1.21 | 1.39 |  | 1.24 | 1.49 | 1.17 | 1.04 | 1.20 | 1.35 | 1.03 |
|  | Yes | 1.41 | 1.16 | 1.73 | 1.19 | 1.35 | 1.52 | 2.10 | 1.24 | 1.18 |  | 1.19 | 2.15 | 1.27 | 1.07 | 1.21 | 1.28 | 1.17 |
| Heart failure | No | 1.43 | 1.10 | 1.83 | 1.07 | 1.17 | 1.32 | 1.45 |  | 1.32 | 1.15 | 1.17 | 1.32 | 1.22 | 1.05 | 1.10 | 1.43 | 1.03 |
|  | Yes | 1.46 | 1.18 | 1.62 | 1.10 | 1.25 | 1.60 | 1.81 |  | 1.43 | 1.17 | 1.38 | 1.89 | 1.16 | 1.08 | 1.06 | 1.38 | 1.15 |
| Type of ICU | TSICU | 1.46 |  | 1.79 | 1.07 | 1.20 | 1.33 | 1.64 | 1.23 | 1.29 | 1.18 | 1.12 | 1.63 | 1.21 | 1.07 | 1.15 | 1.37 | 1.07 |
|  | SICU | 1.42 |  | 2.1 | 1.06 | 1.23 | 1.48 | 1.67 | 1.37 | 1.50 | 1.17 | 1.20 | 1.51 | 1.19 | 1.11 | 1.24 | 1.28 | 1.05 |
